# Attenuation of cerebral ischemia-reperfusion induced neurotoxicity by telmisartan, ertugliflozin, and omaveloxolone through Nrf2/HO-1 pathway modulation: In vivo and in silico insights

**DOI:** 10.1016/j.toxrep.2025.102170

**Published:** 2025-11-19

**Authors:** Yasser J.H. Alyassery, Ahsan F. Bairam, Carlos Medina Martin

**Affiliations:** a Department of Pharmacy Sciences, Faculty of Pharmacy, University of Kufa, Najaf 54001, Iraq; b Department of Pharmacology and Toxicology, Faculty of Pharmacy, University of Kufa, Najaf 54001, Iraq; c School of Pharmacy and Pharmaceutical Sciences, Trinity College Dublin, Dublin 2 D02 PN40, Ireland

**Keywords:** Ischemia-reperfusion injury, Neurotoxicity, Nrf2, HO-1, Telmisartan, Ertugliflozin, Omaveloxolone, Keap1, GSK-3β

## Abstract

Ischemia-reperfusion (IR) injury in the brain is a major cause of brain damage and neurotoxicity, particularly in patients with hypertension and diabetes, where oxidative stress and inflammation play critical roles. This study investigated the effects of telmisartan, ertugliflozin and omaveloxolone, on cerebral ischemia reperfusion IR-induced damage and neurotoxicity through modulation of the nuclear factor erythroid 2–related factor 2/ heme oxygenase-1 (Nrf2/HO-1) signaling pathway. Forty-two rats were divided into seven groups, including controls and treatment groups that received the drugs before ischemia induction via bilateral common carotid artery occlusion (BCCAO) followed by reperfusion. All three agents markedly enhanced Nrf2 immunoreactivity through immunohistochemical analysis and heme oxygenase-1 (HO-1) gene expression in brain tissues compared to controls. They also attenuated neurotoxic outcomes by reducing histopathological damage and lowering inflammatory mediators such as Nuclear Factor kappa-B (NF-κB), Tumor Necrosis Factor-alpha (TNF-α), Interleukin-6 (IL-6), and Matrix Metalloproteinase-9 (MMP-9). A significant negative correlation was observed between Nrf2 activation and the severity of neurotoxicity inflammatory markers. In silico analysis revealed strong binding through highly negative docking scores of telmisartan and ertugliflozin to Nrf2 negative regulators Keap1 and GSK-3β, supported by stable molecular dynamics simulations, suggesting direct inhibition. In conclusion, omaveloxolone, telmisartan, and ertugliflozin alleviate ischemia-reperfusion induced neurotoxicity via potential Nrf2-mediated antioxidant and anti-inflammatory mechanisms, highlighting their potential preventive role in conditions predisposing to stroke.

## Highlights

• Telmisartan, ertugliflozin, and omaveloxolone could activate the Nrf2/HO-1 signaling axis, mitigating cerebral ischemia-reperfusion-induced neurotoxicity.
• All studied pretreatments significantly decrease levels of inflammatory markers in brain tissues.
• Histopathological Improvement, observed reduction in neuronal damage post-treatment.
• In Silico Validation confirm strong binding affinities of telmisartan and ertugliflozin to Nrf2 regulators.
• Findings suggest potential repurposing of these agents for stroke prevention in at-risk populations.

## Graphical Abstract

**Figure:**
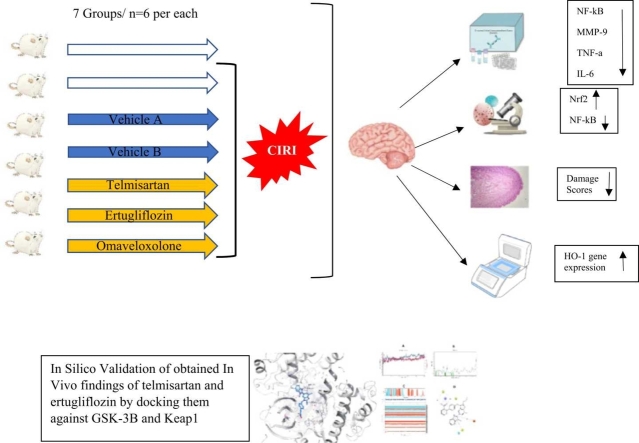

## 1 Introduction

Ischemic stroke, 87 % of all strokes, results from vessel blockage, causing global cerebral ischemia and metabolic injury. Although reperfusion restores cerebral blood flow, it paradoxically induces neurotoxicity manifested by oxidative stress, inflammation, endothelial injury, and blood–brain barrier disruption. During cerebral ischemia-reperfusion injury, hypoxia depletes ATP and generates oxidative stress via Ca²⁺-dependent enzymes, phospholipid breakdown, and mitochondrial dysfunction, overwhelming neuronal antioxidant [1], [2], [3] The transcription factor Nrf2 plays a central role in cellular defense against oxidative and inflammatory neurotoxicity. Nrf2 interacts with regulatory proteins such as Keap1, GSK-3β, and Hrd1, which govern its degradation and stability, while its downstream effector, heme oxygenase-1 (HO-1), serves as a critical antioxidant enzyme [4]. The proteins implicated in Nrf2 regulation include Kelch-like ECH-associated protein 1 (Keap1), a negative regulator of Nrf2 and glycogen synthase kinase-3β (GSK-3β), which phosphorylates Nrf2, facilitating its degradation through β-transducin repeats-containing protein (β-TrCP-Cul1-based ubiquitin ligase); and E3 ubiquitin ligase synoviolin (Hrd1), which is involved in protein degradation within the endoplasmic reticulum [5]. The most important down streaming enzyme which is closely related to Nrf2 state was heme oxygenase-1 (HO-1) [6]. Although HO-1 provides cytoprotective and anti-inflammatory effects, its dual role in disease and cancer progression has been debated. Moreover, the crosstalk between Nrf2 and NF-κB pathways is crucial, since NF-κB regulates inflammatory mediators including TNF-α, IL-6, and MMP-9, which exacerbate neurotoxicity and tissue remodeling [7] [8]. IL-6, TNF-α, and MMP-9 are key players in inflammation and tissue remodeling, linked through signaling pathways like NF-κB and MAPKs (ERK, JNK, p38). MMP-9 breaks down collagen type IV and gelatin in the extracellular matrix, which helps with things like angiogenesis and tissue healing. But its activity may also make things worse, including persistent inflammation and cancer spreading. MMP-9 helps immune cells go to places where there is inflammation by breaking down basement membranes and ECM [9]. Based on the above, targeting Nrf2-mediated suppression of NF-κB-driven inflammation has therefore emerged as a promising toxicological and pharmacological strategy to reduce ischemia-reperfusion–induced neurotoxicity. Moreover, hypertension and diabetes constitute significant risk factors for both ischemic and hemorrhagic strokes [10]. From a pharmacological perspective, some antihypertensive and antidiabetic drugs have demonstrated multifunctional actions. Among these drugs, telmisartan and ertugliflozin demonstrated significant preventive antioxidant effects alongside their therapeutic properties [11], [12]. However, the effects of these drugs on the Nrf2 pathway in the brain during ischemic injury/reperfusion have not been previously studied. Moreover, omaveloxolone received Food and Drug administration FDA approval in 2023 for the treatment of Friedreich's ataxia as a Nrf2 activator [13]. The later agent also may have an impact on prevention and treatment of cerebral ischemia / reperfusion injury. Accordingly, the present study aimed to investigate the ability of telmisartan, ertugliflozin, and omaveloxolone to attenuate CIRI-induced neurotoxicity in a rat bilateral common carotid artery occlusion/reperfusion (BCCAO/R) model. The focus was placed on the Nrf2/HO-1 signaling axis, its regulatory proteins (Keap1, GSK-3β), and the downstream inflammatory mediators NF-κB, TNF-α, IL-6, and MMP-9, alongside histopathological assessments.

## 2 Methods

### 2.1 Preparation of drugs

All drugs were purchased from Macklin Co. China. Telmisartan was prepared as 10 mg/ml stock solution, stored at 4°C, and diluted daily to 1 mg/ml in 10:90 DMSO:corn oil. Ertugliflozin (250 mg) was prepared as a 50 mg/ml stock solution, stored at 4°C, and diluted daily to 5 mg/ml in the same vehicle. Omaveloxolone (50 mg) was dissolved in 5 ml DMSO to a final concentration of 10 mg/ml. Drug dosages were adjusted by animal weight.

### 2.2 Preparation of animals

A total of 42 adult male Sprague Dawley rats, weighing between 210 and 245 g, were used in this study. The animals were housed in the Animal House at the Faculty of Sciences, University of Kufa, under controlled environmental conditions, including a constant temperature of approximately 25°C and a 12-hour light/dark cycle. Food and water were provided *ad libitum* throughout the experiment. Following a 15-day acclimatization period, the animals were randomly assigned to experimental groups in accordance with the study protocol. All procedures were conducted at the Animal Research Center, Faculty of Sciences, and the Laboratory of Pharmacology and Toxicology, Faculty of Pharmacy, University of Kufa.

### 2.3 Ethical considerations

The Institutional Animal Care and Use Committees (IACUCs) and the Central Committee for Bioethics at the University of Kufa authorized this study (Approval No. 13191; May 18th, 2025). All experimental protocols complied with the National Institutes of Health (NIH) Guide for the Care and Use of Laboratory Animals and conformed to worldwide ethical guidelines for the treatment of research animals. The work complies with the ARRIVE principles, highlighting the significance of transparency and reproducibility in animal research [14].

### 2.4 Experimental protocol

Bilateral Common Carotid Artery Occlusions/ Reperfusion (BCCAO/R): All animals were anesthesized with ketamine (80 mg/kg) and xylazine (5 mg/kg) and mantained at 37 °C under a light source during the procedutre. Once anesthetized, each rat was positioned in a supine position on the plate. A midline cervical incision was made to expose the common carotid arteries which then were carefully isolated and occluded using atraumatic vascular clamps to induce cerebral ischaemia. The occlusion was maintained for 30 min, followed by a 60-minute reperfusion period initiated by the removal of the clamps. At the end of the reperfusion phase, all animals were euthanized by decapitation. [15], [16]

The rats were randomly allocated into seven groups after the initial two weeks of optimization, with each group including six rats, as follows [15], [16]:

**Group 1 (sham group):** The rats underwent surgical procedure that mimics the BCCAO but without occluding the arteries. No drugs were given

**Group 2 (control group):** The rats were subjected to BCCAO/R No drugs were given.

**Group 3 (vehicle group A):** The rats received a daily oral dose of DMSO plus corn oil (10:90) for one week, followed by BCCAO/R.

**Group 4 (vehicle group B):** The animals received a daily intraperitoneal (IP) dose of DMSO for two days prior to undergoing BCCAO/R.

**Group 5 (Telmisartan group):** Rats received a daily oral dose of telmisartan (3 mg/kg/day) in DMSO + corn oil (10:90) for one week, followed by BCCAO/R.

**Group 6 (Ertugliflozin group):** Rats received a daily oral dose of ertugliflozin (20 mg/kg/day) in DMSO + corn oil (10:90) for one week, followed by BCCAO/R.

**Group 7 (Omaveloxolone group):** Rats were given a daily IP dose of omaveloxolone (10 mg/kg) in DMSO for two days, followed by BCCAO/R.

The selected dosages of telmisartan (3 mg/kg), ertugliflozin (20 mg/kg), and omaveloxolone (10 mg/kg) were based on previously published studies demonstrating their efficacy in modulating inflammatory and oxidative stress pathways without inducing adverse effects. The telmisartan dose was chosen considering its favorable bioavailability and well-documented pleiotropic anti-inflammatory effects in preclinical models, particularly within the central nervous system (CNS), while avoiding hypotensive effects that could complicate stroke management [17], [18], [19], [20], [21], [22]. Similarly, the dosage of ertugliflozin was selected based on prior studies highlighting its protective, non-glycemic pleiotropic actions [23], [24]. For omaveloxolone, the selected dose was informed by recent evidence supporting its neuroprotective efficacy and its emerging clinical role as an NRF2 activator in the treatment of neurological disorders [25].

### 2.5 Preparation of brain tissue samples

Following decapitation, the brain was carefully extracted by dissecting the skull from the foramen magnum posteriorly. The olfactory bulbs and cerebellum were subsequently removed. The midbrain and forebrain were then rinsed with ice-cold phosphate-buffered saline (PBS) and kept on ice to preserve tissue integrity for further experimental procedures. The brain tissue was coronally sectioned into segments, with one portion fixed in 10 % formalin solution for immunohistochemistry (IHC) and histopathological investigations. The formalin was refreshed after the initial 24 h to ensure optimal fixation. The second portion was placed in TRIzol reagent-filled Eppendorf tubes and immediately stored at −80°C for subsequent quantitative real-time PCR (qRT-PCR) analysis. While segments designated for ELISA were weighed, washed with PBS, placed in sterile Eppendorf tubes, flash-frozen on dry ice, and stored at –80°C until analysis.

### 2.6 Immunohistochemical analysis

Immunohistochemical analysis was performed to evaluate the expression of Nrf2 and NF-κB in rat brain tissue samples. Tissue sections were incubated with Rabbit Anti-Nrf2 antibody (Cat. No. SL1074R) and Rabbit Anti-NF-κB p65 antibody (Cat. No. 20159 R), both obtained from Sunlong Biotech, China. For specificity assessment, negative controls were included by processing parallel sections without the primary antibody to exclude non-specific staining and background signal. This procedure enabled accurate visualization and evaluation of Nrf2 and NF-κB expression in the studied brain tissues. 3 µm brain tissue sections on charged slides were deparaffinized with xylene (15 min) and dehydrated through graded ethanol. Antigen retrieval was performed using Tris-EDTA buffer under heat and pressure. Endogenous peroxidase activity was blocked by incubating with blocking solution (100 µl) for 15 min at room temperature in a dark, humid chamber. Slides were incubated overnight at 4°C with primary antibody (1:200 dilution). Detection was done using a peroxidase-linked polymer secondary kit (100 µl, 30 min at room temperature), followed by DAB substrate incubation (100 µl, 10 min in dark). Counterstaining was done with hematoxylin for 2 min, rinsed, then dehydrated again through ethanol series and cleared in xylene (5 min). Slides were dried and stored until analysis. Scoring was performed by one blinded researcher within two weeks for consistency [26]. The interpretation of immunoreactivity relies on the histochemical scoring (H-score) assessment, which integrates both staining intensity (i) and the percentage of stained cells at each intensity level (Pi). The i values are classified as follows: 0 indicates no evidence of staining, 1 denotes weak staining, 2 represents moderate staining, and 3 signifies strong staining. The values of Pi range from 0 % to 100 %. The final H-score is calculated as the sum of i multiplied by Pi. The score ranges from 0 to 300 [27], [28].

### 2.7 Histopathological examination

Brain tissues fixed in 10 % formalin were dehydrated through graded alcohols (60–100 %), cleared with xylene, and embedded in paraffin. Thin horizontal Section (5 μm) were stained with Hematoxylin and Eosin and examined by a blinded pathologist [29]. Histological damage was scored on a scale from 0 to 3, where Score 1 indicates modest alterations, characterized by the presence of edema, pyknotic neurons, eosinophils, and/or darkened (atrophied) shrunken eosinophilic neurons. Score 2 denotes moderate alterations in histological tissues, characterized by a minimum of two hemorrhagic regions. Score 3 indicates significant alterations in histological tissues, characterized by distinct infarcted foci or localized necrosis [30], [31].

### 2.8 Hmox1 mRNA expression in brain tissue samples

The Real Time PCR primers for quantification of heme oxygenase 1 (*Hmox1*) gene and normalized by GAPDH housekeeping gene. These primers were design by using NCBI-GenBank and primer3 plus design and provided by (Macrogen company, Korea) as shown in Table 1. Relative gene expression was quantified using the 2^(-ΔΔCt) method, ensuring accurate comparison of target gene expression levels across different samples. [32]

**Table 1:** The primers used for q RT-qPCR with their sequences, product size, and amplicon.

**Primer**	**Sequence (5′-3′)**		**Product size**	**Amplicon** **)NCBI Ref.(**
Rattus *Hmox1*, mRNA gene	F	AGAGCGAAACAAGCAGAACC	137 bp	NM_012580.2
	R	TGGCTGGTGTGTAAGGGATG		
Rattus *GAPDH*, mRNA gene	F	CCCTCAAGATTGTCAGCAATGC	134 bp	NM_017008.4
	R	AGTCTTCTGAGTGGCAGTGATG		

### 2.9 Measurement of inflammatory biomarker levels

According to manufacture instructions, the levels of NF-kB (Cat. No. SL0537Ra), TNF-α (Cat. No. SL0722Ra), and MMP-9 (Cat. No. SL0490Ra) were estimated using ELISA kits obtained from Sunlong Biotech Co., Ltd, China, while IL-6 (Cat. No. E0135Ra) was estimated using ELISA kit obtained from Bioassay Technology Lab., China.

### 2.10 Molecular Docking Study

GLIDE (Grid-Based Ligand Docking with Energetics) is a prominent molecular docking software created by Schrödinger. The LigPrep application was utilized for the preparation of TELMISARTAN_CID_65999 and ERTUGLIFLOZIN_CID_44814423. The technique involved the introduction of hydrogen atoms, the removal of ionic compounds, and the incorporation of ions within a pH range of 7 ± 2.0. Crystal structures of Keap1 (pdb: 7OFE) and GSK-3β (pdb: 5K5N) were first downloaded from PDB. The protein preparation module was employed to generate the three-dimensional structures of both native and mutant protein variations. This entailed the completion of missing side chains, insertion of cap termini, formation of disulfide bonds, inclusion of hydrogen molecules, and establishment of bond ordering [33]. The process of minimizing energy was executed utilizing the OPLS3e force field [34], [35]. A grid box was created utilizing the known binding locations of the proteins. The Receptor Grid Generation module utilized default settings of a van der Waals perimeter scaling factor of 1.0 and a charging threshold of 0.25 to ascertain the grid dimensions. The Ligand Docking program and the GLIDE XP parameters were used to carry out the GLIDE docking. Prior to the analysis of medicinal compounds, a scaling factor of 0.80 was used to adjust the van der Waals radius, and a score threshold of 0.15 was employed as a standard criterion (Khan *et al.,* 2023). Docking score = avdW + bCoul + Hbond + Metal + Lipo + BuryP + RotB + Site [36]. The present context employs the variables 'a' and 'b' to represent the coefficient standards of van der Waals (vdW) and Coulomb (Coul) energies, correspondingly. In addition, the term 'vdW' denotes the van der Waals energy, while 'Coul' is indicative of the Coulomb energy. Both 'Hbond' and 'Metal' refer to the protein's ability to form hydrogen bonds, while 'Association with Metal' emphasizes this property. 'Lipo' stands for the usual expression of lipophilicity, and 'BuryP' is shorthand for the penalty for a buried polar group. Assigning a letter to the penalty for a rotatable bond ('RotB') and describing polar interactions at the active site [36].

### 2.11 Molecular dynamic study

Protein-ligand interactions at the atomic scale and conformational alterations during the binding process were analyzed by a 100 ns molecular dynamics simulation employing Newton's equations of motion via Desmond from the Schrödinger suite [37]. All four complexes underwent preprocessing utilizing Maestro's protein preparation wizard module, systems were constructed with the "system builder tool" and the OPLS5e force field employing default settings [38]. The energy of both systems must be reduced prior to achieving equilibrium in the orthorhombic box (10 Å×10 Å×10 Å) of the TIP3P water model. This is achieved by heating to 300 K at 1 atm pressure and including 0.15 M NaCl. Receptor-ligand interactions were examined using a simulated interaction diagram tool. [39]

### 2.12 Statistical analysis

Data analysis was performed using GraphPad Prism version 9.0.0 (GraphPad Software, La Jolla, California, USA) for Microsoft Windows. Results are presented as (means ± SD). Data distribution normality was assessed using the Shapiro-Wilk test. For normally distributed data, one-way ANOVA followed by Tukey’s HSD post hoc test was conducted. Fisher-Freeman-Halton test was used for histopathological damage score overall comparisons among experimental groups. Kruskal-Wallis’s test was employed for overall group comparisons also and then post hoc Dunn’s test was subsequently used for pairwise multiple comparisons based on median values. Pearson correlation analysis performed between Nrf2 expression and the levels of inflammatory markers as well as between Nrf2 expression and histological scores. Statistical significance was set at P < 0.05.

## 3 Results

### 3.1 Total Nrf2 and nuclear Nrf2 immunoreactivity

**Fig. 1:**
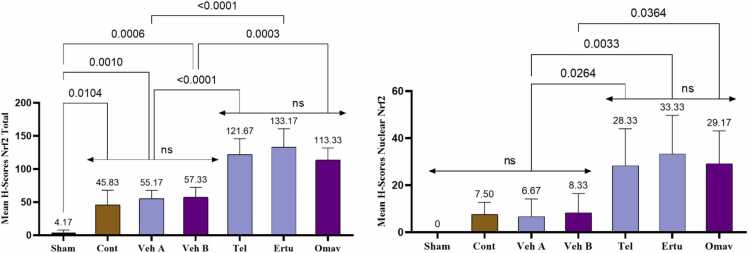
H-scores for Nrf2 total and nuclear immunoreactivity in brain tissues. Mean values are displayed above each bar, and p-values are indicated along the pairwise comparison lines. Cont: Control, Veh A: Vehicle A (DMSO + Corn Oil PO), Veh B: Vehicle B (DMSO IP), Tel: Telmisartan, Ertu: Ertugliflozin, Omav: Omaveloxolone, ns: no significant difference.

**Fig. 2:**
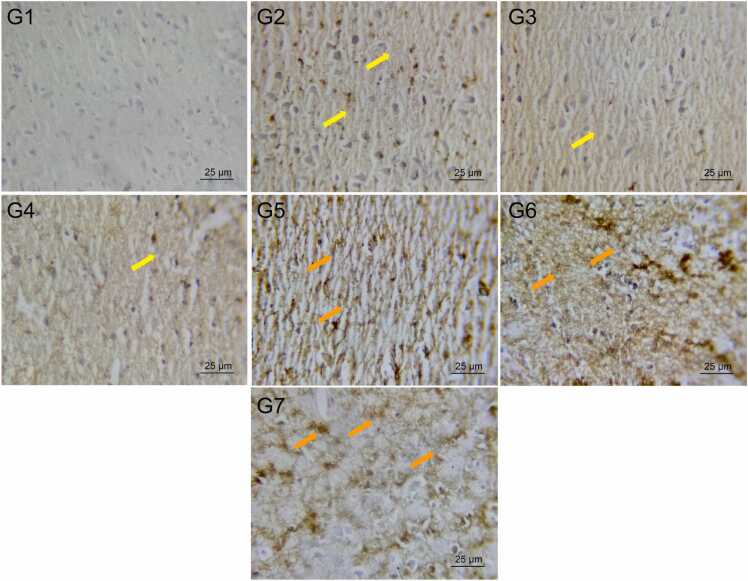
Optical microscope images demonstrate Nrf2 immunoreactivity in rat brain cortical tissues across the seven experimental groups. Group 1 (sham) exhibits negative immunoreactivity. Groups 2–4 display weak positive immunoreactivity (yellow arrows), whereas Groups 5–7 show moderate positive Nrf2 immunoreactivity (orange arrows), as detected by immunohistochemical analysis.

The total Nrf2 immunoreactivity was significantly elevated in animals treated with telmisartan and ertugliflozin (Groups 5 and 6, respectively) compared with their corresponding vehicle-administering animals (Group 3, vehicle A). Similarly, omaveloxolone-administered animals (Group 7) showed a significant increase in total Nrf2 immunoreactivity compared with group 4 (vehicle B). However, no significant differences were seen among groups 5, 6, and 7. Furthermore, animals in groups 2 (control), 3 (vehicle A), and 4 (vehicle B) demonstrated raised levels of total Nrf2 immunoreactivity compared to those in group 1 (sham), with no significant differences among these corresponding groups, as shown in Fig. 1, Fig. 2.

There were no significant differences in Nrf2 nuclear immunoreactivity among Group 1 (sham), Group 2 (control), Group 3 (vehicle A), and Group 4 (vehicle B). Groups 5 (telmisartan) and 6 (ertugliflozin) showed a significant increase in Nrf2 nuclear immunoreactivity compared with their corresponding vehicle-administering animals (Group 3, vehicle A), whereas Group 7 (omaveloxolone) exhibited a significant increase compared to Group 4 (vehicle B). No significant differences were observed among Groups 5, 6, and 7. These results are presented in Fig. 1, Fig. 2.

### 3.2 Heme Oxygenase - 1 (*Hmox1*) gene expression

**Fig. 3:**
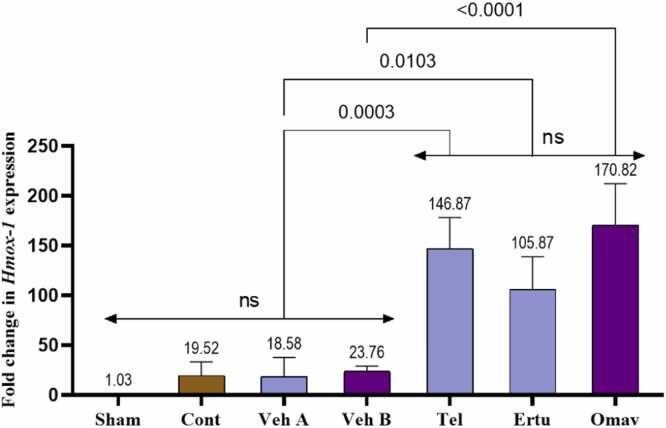
Mean ± SD fold change in Hmox-1 gene expression in brain tissues. Mean values are displayed above each bar, and p-values are indicated along the pairwise comparison lines. Cont: Control, Veh A: Vehicle A (DMSO + Corn Oil PO), Veh B: Vehicle B (DMSO IP), Tel: Telmisartan, Ertu: Ertugliflozin, Omav: Omaveloxolone, ns: no significant difference.

The increase in Hmox1 gene expression observed in Groups 2 (control), 3 (vehicle A), and 4 (vehicle B) was not statistically significant compared to Group 1 (sham). However, Hmox1 expression was significantly upregulated in Group 5 (telmisartan) and Group 6 (ertugliflozin) compared to Group 3 (vehicle A), and in Group 7 (omaveloxolone) compared to Group 4 (vehicle B). No significant differences were detected among Groups 5, 6, and 7 (Fig. 3).

### 3.3 Inflammatory markers

**Fig. 4:**
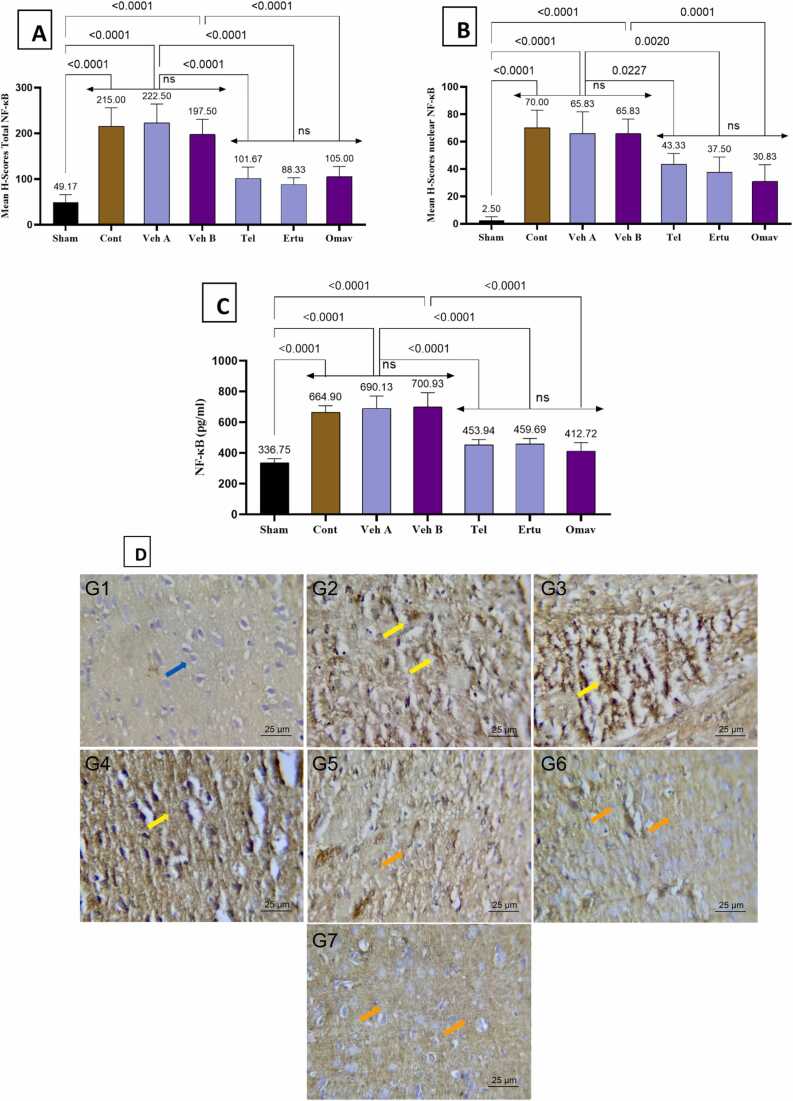
Mean H-scores for (A) NF-κB total immunoreactivity (B) NF-κB nuclear immunoreactivity in brain tissues, (C) Brain tissues NF-κB levels pg/ml. (D) Optical microscope images demonstrate NF-κB immunoreactivity in rat brain cortical tissues across the seven experimental groups. Mean values are displayed above each bar, and p-values are indicated along the pairwise comparison lines. Cont: Control, Veh A: Vehicle A (DMSO + Corn Oil PO), Veh B: Vehicle B (DMSO IP), Tel: Telmisartan, Ertu: Ertugliflozin, Omav: Omaveloxolone, ns: no significant difference. positive immunoreactivity appears in brown DAB-stained areas while negative areas counterstained by hematoxylin (blue arrow). Moderate to strong DAB staining intensity (yellow arrows) appear in control and vehicle groups. Groups pretreated with either telmisartan or ertugliflozin or omaveloxolone has been show slight DAB staining intensity (orange arrows).

**Fig. 5:**
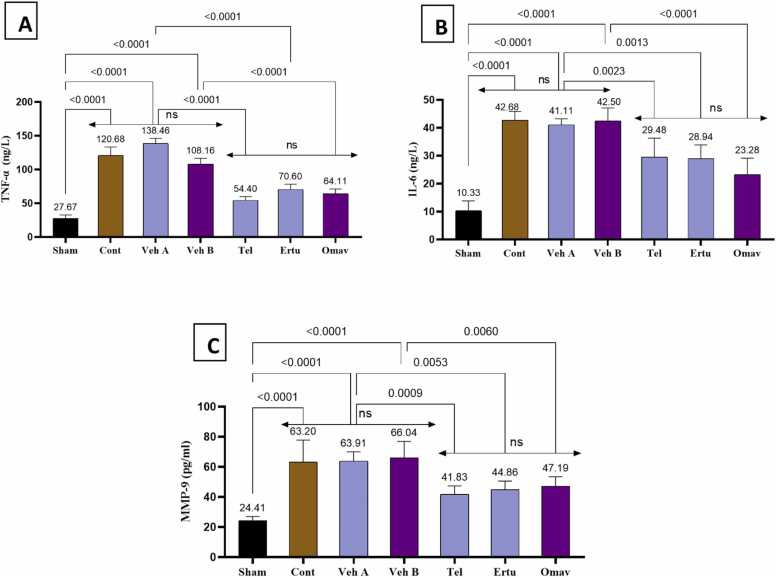
Brain tissue (A) TNF-α levels (ng/L), (B) IL-6 levels (ng/L), (C) MMP-9 levels (pg/ml). Mean values are displayed above each bar, and p-values are indicated along the pairwise comparison lines. Cont: Control, Veh A: Vehicle A (DMSO + Corn Oil PO), Veh B: Vehicle B (DMSO IP), Tel: Telmisartan, Ertu: Ertugliflozin, Omav: Omaveloxolone, ns: no significant difference.

This study shows that induction of brain injury by BCCAO, followed by a reperfusion period, in group 2 (control), has shown a significantly increase NF-kB immunoreactivity (total and nuclear) as compared to the sham group (Fig. 4). Similarly, group 2 shows a significant increase in TNF-α, MMP-9, and IL-6 levels compared to sham (Fig. 5). Pretreatment with either telmisartan, ertugliflozin, or omaveloxolone significantly downregulated all inflammatory biomarkers level when compared to their corresponding vehicles (Fig. 4, Fig. 5).

### 3.4 Histopathological findings

**Fig. 6:**
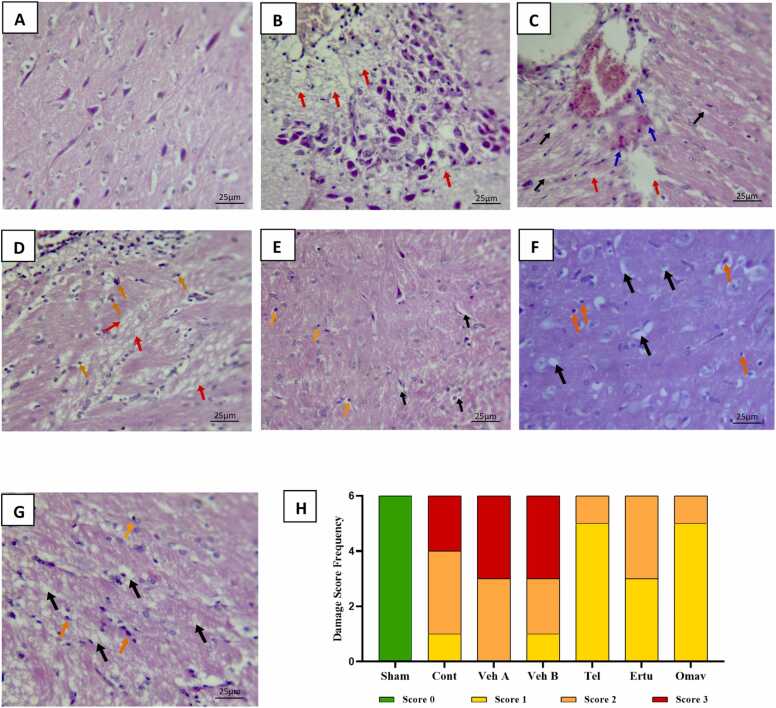
**Photomicrographs of a cortical brain section from all experimental groups with frequency distribution of histopathological damage scores.***Magnification power (400 ×). Dark, shrunken, and eosinophilic neurons (orange arrows), oedema (black arrows), hemorrhage (blue arrows), and focal infarctive lesions (red arrows). Sham groups show normal tissue architecture with normal spindle shapes of neurons, while control and vehicle groups show different features of histopathological damage and loss of normal tissue architecture by diffuse sites of hemorrhage, infarcted areas, in addition to infiltration of eosinophils and other inflammatory cells and most of neurons turn to dark and shows pyknosis. In groups pretreated with either telmisartan, ertugliflozin, and omaveloxolone, most of findings correlate with damage score of 1 such as pyknotic neurons and edema with absent of infarcted area and limited areas of hemorrhages within sections. A: Sham, B: Control, C: Vehicle A (DMSO + Corn Oil PO), D: Vehicle B (DMSO IP), E: Telmisartan, F: Ertugliflozin, G: Omaveloxolone, H: The frequency distribution of histopathological damage scores among the study groups.*

The Fisher–Freeman–Halton test revealed a highly significant difference in histopathological damage scores among the seven groups (p < 0.01). In the sham group (G1), all brain sections exhibited normal architecture without detectable damage. In contrast, the control (G2), vehicle A (G3), and vehicle B (G4) groups displayed severe neuropathological alterations, including extensive areas of pyknotic eosinophilic neurons, multiple hemorrhagic foci with extravasation, and well-defined necrotic regions. Several samples in these groups reached the maximum damage score of 3. Pre-treatment with telmisartan (G5), ertugliflozin (G6), or omaveloxolone (G7) markedly attenuated histopathological injury. Most samples from these groups showed only mild alterations (damage score 1), characterized mainly by scattered pyknotic neurons and limited eosinophilic infiltrations. Importantly, hemorrhagic lesions were minimal and infarcted areas were absent in the majority of examined sections (Fig. 6).

**Correlation between Nrf2 immunoreactivity and Inflammatory markers of the study and histopathological damage scores**

**Fig. 7:**
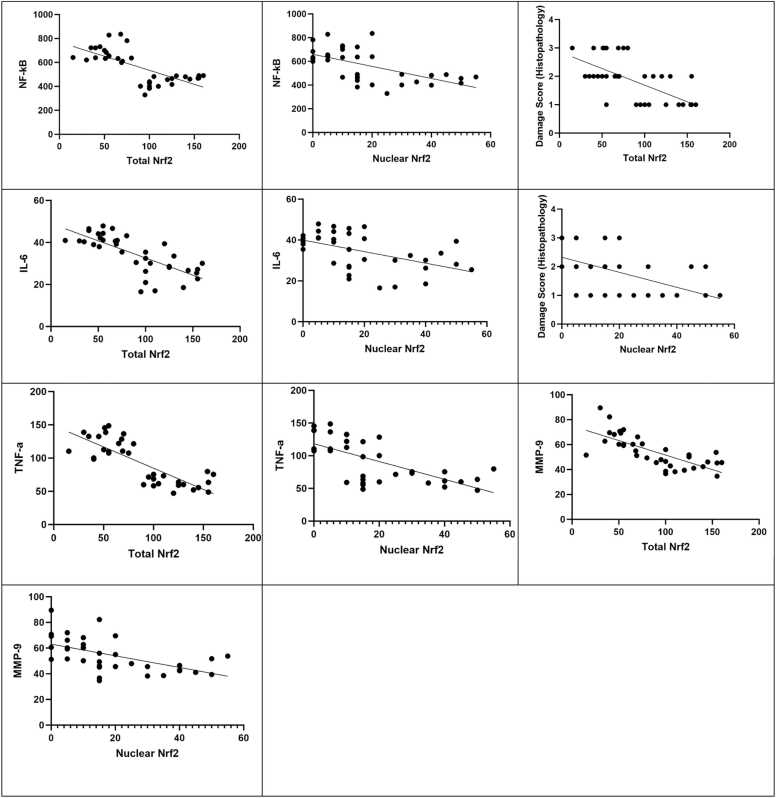
Pearson correlation graphs with linear regression lines between Nrf2 immunoreactivity (Total and Nuclear) and Inflammatory markers of the study and histopathological damage scores.

In the present study, Pearson’s correlation analysis was performed to evaluate the relationship between Nrf2 expression and inflammatory responses in the disease model following pretreatment with the investigated agents. The analysis revealed a clear negative correlation between Nrf2 immunoreactivity and all inflammatory markers (Fig. 7).

Molecular **Docking of Telmisartan and Ertugliflozin against Nrf2 Negative Regulators Keap1 and GSK-3β**

**Fig. 8:**
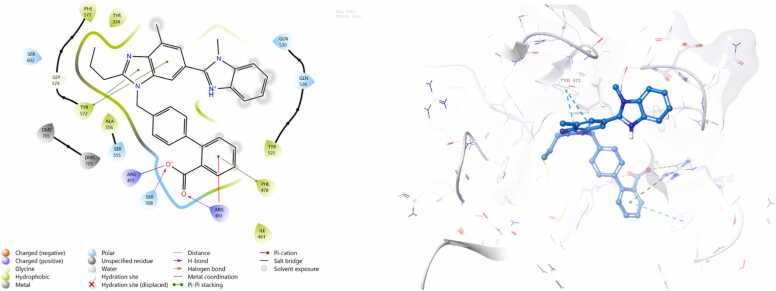
Schematic 2D and 3D representation of telmisartan binding mode within the active site of Keap1 (pdb:7ofe). Surface map shows that telmisartan fits well in the binding pocket of Keap1. active site contour represented in grey colour whereas ligand appear in azure.

**Fig. 9:**
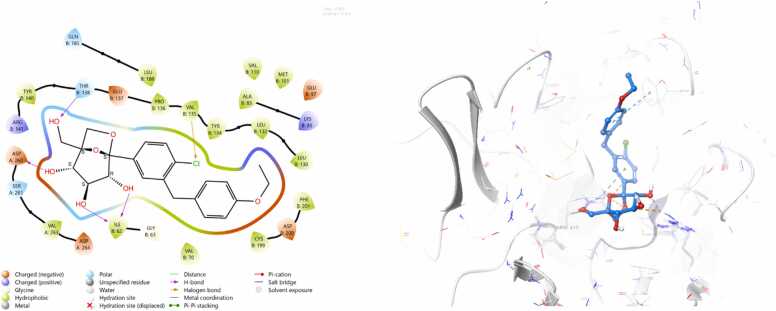
Schematic 2D and 3D representation of ertugliflozin binding mode within the active site of Keap1 (pdb:7ofe). Surface map shows that ertugliflozin fits well in the binding pocket of Keap1. active site contour represented in grey colour whereas ligand appear in azure.

**Fig. 10:**
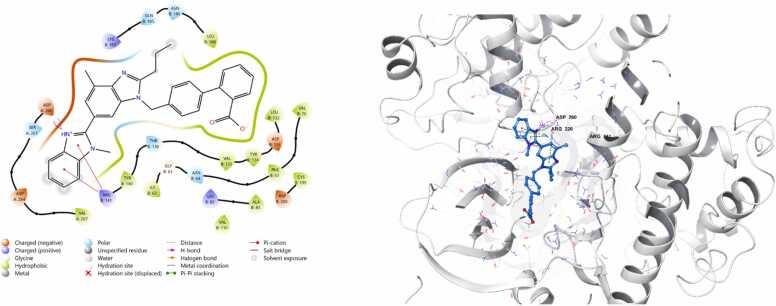
Schematic 2D and 3D representation of telmisartan binding mode within the active site of GSK3b (pdb:5k5n). Surface map shows that telmisartan fits well in the binding pocket of GSK-3β. active site contour represented in grey colour whereas ligand appear in azure.

**Fig. 11:**
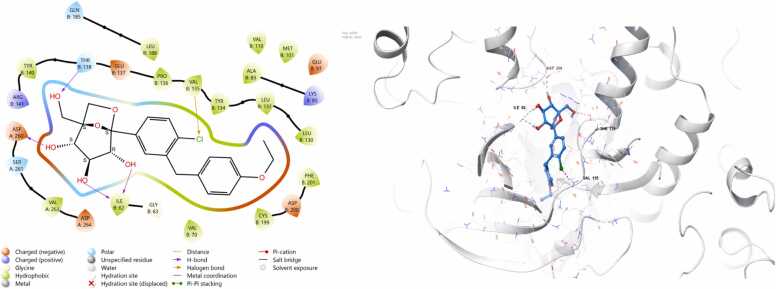
Schematic 2D and 3D representation of ertugliflozin binding mode within the active site of GSK3b (pdb:5k5n). Surface map shows that ertugliflozin fits well in the binding pocket of GSK-3β. active site contour represented in grey colour whereas ligand appear in azure.

*In silico* work was intentionally conducted after obtaining interesting in vivo results serving as a complementary mechanistic approach to support and interpret the experimental findings. It was performed only for telmisartan and ertugliflozin, as their potential to activate Nrf2 remains less clearly defined. Docking against Nrf2 negative regulators was therefore essential to provide mechanistic insight into their observed biological effects. In contrast, omaveloxolone is already an FDA-approved and clinically established Nrf2 activator with a well-characterized mechanism of action (Covalent Inhibitor, Electrophile). The docking protocol was validated by superimposing the redocked ligands onto their respective crystallized conformations. The calculated root mean square deviations (RMSD) were 0.646 Å for Keap1 and 0.804 Å for GSK-3β, both of which fall well within the acceptable threshold of ≤ 2 Å, thereby confirming the reliability of the docking procedure. Both telmisartan and ertugliflozin gives negative docking scores when docked against target Nrf2 regulators as demonstrated in Table 2. The 3D and 2D complexes are shown in (Fig. 8, Fig. 9, Fig. 10, Fig. 11).

**Table 2:** Docking scores of the docked complexes.

**Protein Name**	**PDB ID**	**Ligand**	**Interacting Residue** **(Chain: Residue)**	**Bond Type**	**Docking Score (kcal/mol)**
GSK-3β	5K5N	TELMISARTAN_CID_65999	ARG B:141	Pi-cation	**-7.3012**
GSK-3β	5K5N	ERTUGLIFLOZIN_CID_44814423	THR B:138, ASP A:260, ILE B:62, VAL B:135	H-bond, Halogen bond	**-8.9163**
Keap1	7OFE	TELMISARTAN_CID_65999	TYR572, ARG415, SER508, ARG483, PHE478	H-bond, Salt bridge, Pi-Pi stacking	**-7.7412**
Keap1	7OFE	ERTUGLIFLOZIN_CID_44814423	ARG415, ARG483, TYR572	H-bond, Salt bridge, Pi-Pi stacking	**-8.4122**

**Molecular Dynamic study of Telmisartan and Ertugliflozin against Nrf2 Negative Regulators Keap1 and GSK-3β**

**Fig. 12:**
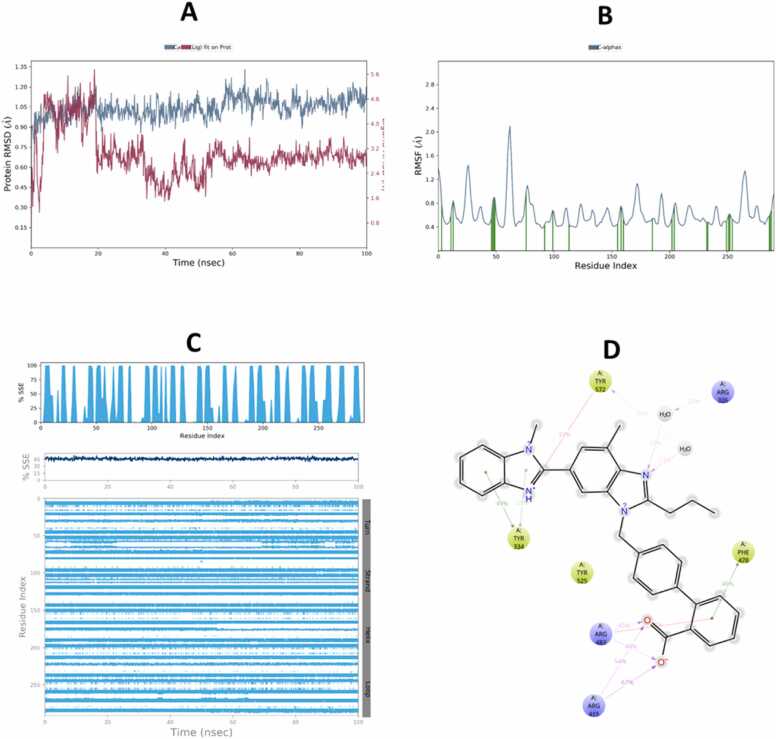
Molecular Dynamic Simulation of telmisartan and Keap1. A: RMSD over the simulation time. The right Y-axis displays ligand variation (red lines), while the left Y-axis displays variation of protein RMSD (blue lines). B: RMSF. C: represent distribution of SSE (beta-strands in blue) across the protein structure by residue index and there was no considerable change in SSE%. D: schematic representation of various interactions between ligand atoms and protein residues.

**Fig. 13:**
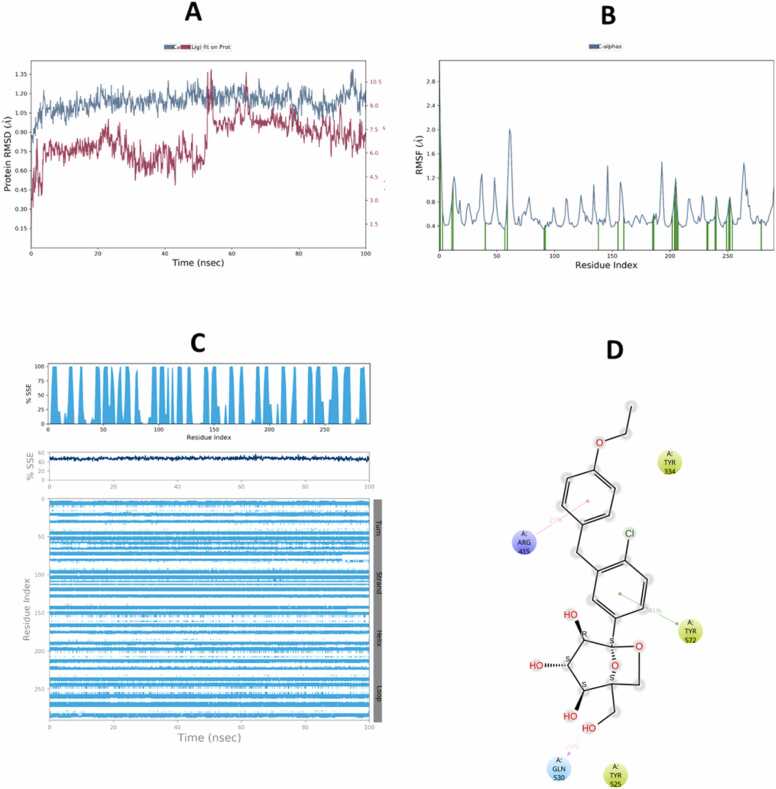
Molecular Dynamic Simulation of ertugliflozin and Keap1. A: RMSD over the simulation time. The right Y-axis displays ligand variation (red lines), while the left Y-axis displays variation of protein RMSD (blue lines). B: RMSF. C: represent distribution of SSE (beta-strands in blue) across the protein structure by residue index and there was no considerable change in SSE%. D: schematic representation of various interactions between ligand atoms and protein residues.

**Fig. 14:**
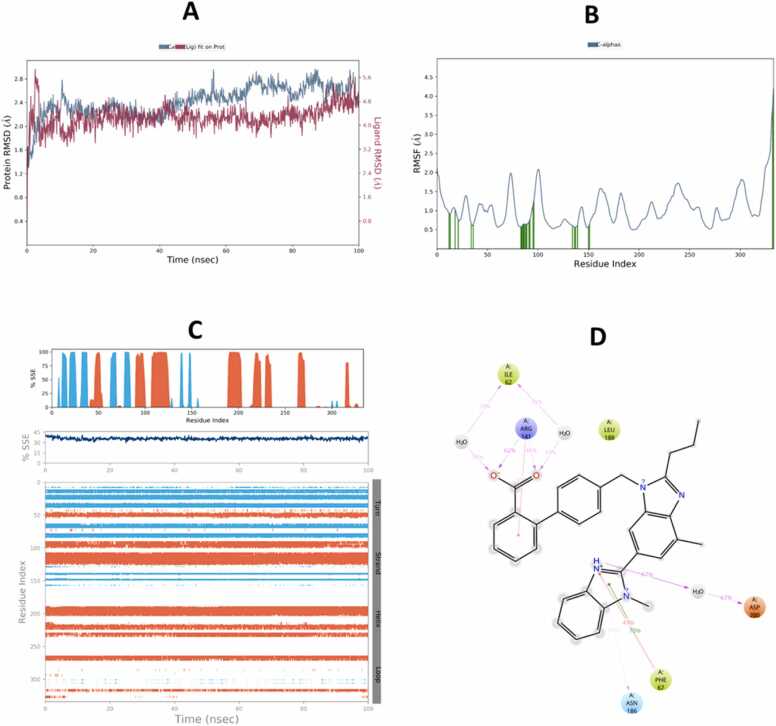
Molecular Dynamic Simulation of telmisartan and GSK-3β. A: RMSD over the simulation time. The right Y-axis displays ligand variation (red lines), while the left Y-axis displays variation of protein RMSD (blue lines). B: RMSF. C: represent distribution of SSE (alpha-helices appear in orange while beta-strands in blue) across the protein structure by residue index and there was no considerable change in SSE%. D: schematic representation of various interactions between ligand atoms and protein residues.

**Fig. 15:**
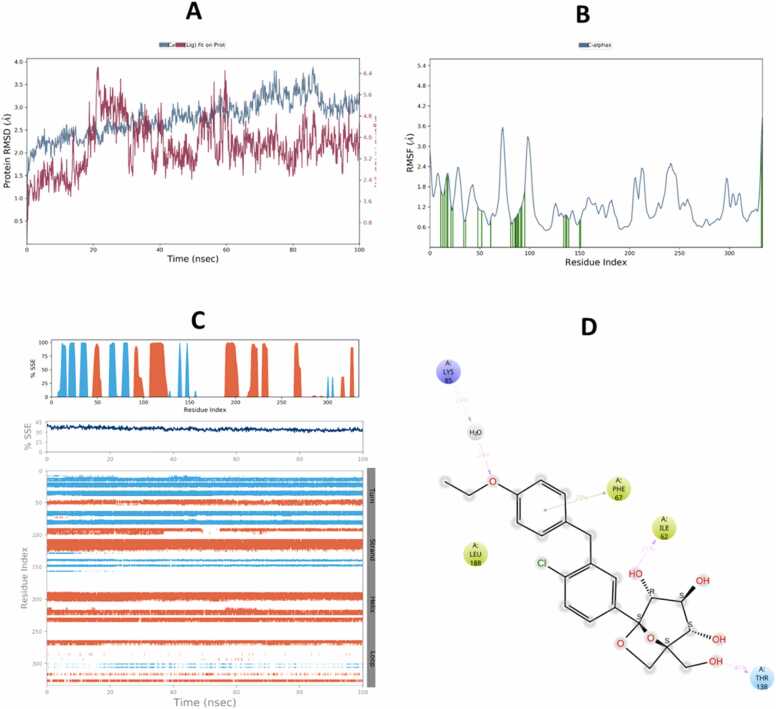
Molecular Dynamic Simulation of ertugliflozin and GSK-3β. A: RMSD over the simulation time. The right Y-axis displays ligand variation (red lines), while the left Y-axis displays variation of protein RMSD (blue lines). B: RMSF. C: represent distribution of SSE (alpha-helices appear in orange while beta-strands in blue) across the protein structure by residue index and there was no considerable change in SSE%. D: schematic representation of various interactions between ligand atoms and protein residues.

The molecular dynamics (MD) simulation results demonstrated stable binding interactions between telmisartan or ertugliflozin and both Keap1 and GSK-3β over a 100 ns simulation period, as illustrated in the corresponding (Fig. 12, Fig. 13, Fig. 14, Fig. 15).

## 4 Discussion

Cerebral ischemia/reperfusion injury (CIRI) triggers a cascade of pathological neurotoxic events involving oxidative stress, inflammation, and structural brain damage, highlighting the importance of targeting molecular and inflammatory pathways as a strategy for neuroprotection in ischemic stroke. BCCAO/R model rather than MCAO were employed because the primary aim was to investigate mechanistic pathways of oxidative stress and inflammation, particularly Nrf2/HO-1 and NF-κB modulation. BCCAO produces a reproducible global ischemic insult with robust oxidative and inflammatory responses across brain tissues, which is advantageous for molecular and histological neurotoxic analyses.

Nrf2 immunoreactivity was largely negative in the sham group, consistent with its low baseline expression due to Keap1-mediated cytoplasmic degradation under normal physiological conditions [40]. Previous research has indicated that healthy brain areas include scarce Nrf2 positive cells, therefore our results are in line with that [41]. Total Nrf2 immunoreactivity was significantly elevated in the control, vehicle A, and vehicle B groups compared to the sham group, while nuclear expression remained unchanged. This suggests increased Nrf2 expression due to CIRI-induced oxidative stress, with impaired nuclear translocation, supporting previous findings on Nrf2 activation in MCAO-induced CIRI [42]. These findings are consistent with previous studies and further supported by evidence of Nrf2 activation in other organs, such as the myocardium, following ischemia-reperfusion injury [43]. Excess ROS, such as superoxide and H₂O₂, modify cysteine residues on Keap1, inducing a conformational change that impairs its ability to degrade Nrf2, leading to Nrf2 accumulation. While this activation serves as a protective response, sustained or excessive ROS can overwhelm cellular defenses, resulting in damage or cell death [44], [45].

The telmisartan-treated group showed a significant increase in both total and nuclear Nrf2 immunoreactivity, indicating activation of the Nrf2 pathway. Similarly, ertugliflozin and omaveloxolone treatment resulted in a significant upregulation of total and nuclear Nrf2 expression further supporting Nrf2 pathway activation. Among angiotensin receptor blockers (ARBs), telmisartan is notable for its partial agonistic activity on PPAR-γ. Some studies suggest that PPAR-γ activation may indirectly influence Nrf2 signaling by modulating the cellular redox state and inflammatory pathways, thereby facilitating the release of Nrf2 from Keap1 and its subsequent translocation into the nucleus [46]. This finding is consistent with previous studies demonstrating that telmisartan enhances Nrf2 expression to alleviate alcohol-induced liver injury [11] as well as Nrf2 activation produced by telmisartan which protect neurons against demyelination by cuprizone in model of multiple sclerosis [47]. Ertugliflozin activates Nrf2 signaling in the liver of mice with thioacetamide-induced liver fibrosis [48]. Possible mechanisms by which Nrf2 is activated by ertugliflozin include that it may stabilize and activate Nrf2 by lowering hyperglycemia, which in turn reduces oxidative stress [49]. Also use of SGLT2 inhibitors has been shown to enhance mitochondrial activity and decrease ROS generation [50]. findings also agree with previous researches in which omaveloxolone mitigate brain endothelial neuroinflammation by activation of Nrf2 [51]. Additionaly, the results of this study corroborate prior research showing that omaveloxolone is effective in treating Alzheimer's disease that produced neuroprotective actions include reducing inflammation in the brain and preventing cell death via the activation of Nrf2 [25]. The main mechanism by which omaveloxolone increases Nrf2 cellular levels is via suppressing Keap1. Nrf2 is able to translocate into the nucleus and stimulate the production of many cytoprotective genes because of its stability [52].

Despite visual increases in fold changes of heme oxygenase-1 gene expression in groups; control, vehicle A, and vehicle B as compared with sham group; these increases are statistically not significant. This is consistent with the findings that nuclear Nrf2 immunoreactivity is not statistically significant among the four groups. This result is in line with other prior investigations [53], [54]. An external activator is necessary to activate the Nrf2 signaling pathway and, by extension, the gene expression of the protective downstream antioxidant enzyme HO-1, which is need sufficient upregulation in an oxidative stress state. Telmisartan, ertugliflozin, and omaveloxolone treatments each significantly upregulated HO-1 gene expression in brain tissue compared to their respective vehicle groups, indicating enhanced antioxidant protection. This aligns with previous findings where telmisartan ameliorated neuropathies by increasing HO-1 levels through Nrf2 pathway activation, as evidenced by elevated Nrf2 and HO-1 expression in the kidneys of both healthy and diabetic rats (Antar et al.*,* 2022). Previous studies have shown that other SGLT2 inhibitors also significantly affect HO-1 expression. For example, empagliflozin activates HO-1 in vascular smooth muscle cells via the Nrf2 pathway [55]. This finding also supports previous studies showing omaveloxolone reduces brain damage by activating the Keap1-Nrf2-ARE pathway [13].

This study shows that induction of CIRI in group 2 (control), group 3 (vehicle A), and group 4 (vehicle B) causes significant increase in NF-κB immunoreactivity (both total and nuclear) and ELISA measured concentrations of inflammatory markers (TNF-a, IL-6, MMP-9, and NF-kB) in comparison to healthy group 1 (sham) and provides robust view of activation of inflammatory response. This result is in line with many previous studies [56], [57], [58]. Pretreatment with either telmisartan, ertugliflozin, or omaveloxolone causes a significant decline in NF-κB levels and inflammatory parameter levels (TNF-α, IL-6, and MMP-9) as compared to their related vehicle groups. This study supports the anti-inflammatory action of telmisartan in a previous studies [59], [60], [61], [62], [63], [64]. Anti-inflammatory finding agrees with previous work that showed ertugliflozin significantly decreased IL-6 and TNF-a levels in mice exposed to endotoxemia [24]. Similarly, previous study showed that ertugliflozin treated groups at different concentrations significantly lowers IL-6 and TNF-a in liver fibrosis model [48]. Besides that, findings lend credence to the idea that omaveloxolone has an anti-inflammatory effect on brain tissues. This finding is in alignment with previous work that showed a significant decline in NF-κB levels in groups treated with omaveloxolone in comparison to controls that were subject to oxidative stress in astrocytes of the rat brain [65].This result agree with previous work that showed decreased neuroinflammation as a result of omaveloxolone treatment both in vivo and in vitro study and gave significant downregulation in TNF-a and IL-6 gene expression [66]. This effect also in line with previous work that showed treatment with omaveloxolone significantly decreased MMP-9 levels in rat model of COPD and this finding correlated with activation of Nrf2 signaling pathway and inhibition of NFkB according to the tested parameters [67], [68].

Brain tissues were evaluated for the impact of CIRI damage using histopathological rating. Nuclear pyknosis and morphological abnormalities are examples of cellular changes that may be revealed by histopathological investigation. Necrotic tissues and intercellular spaces are examples of tissue-level modifications. In this study, global cerebral I/R injury resulted in mild to severe cerebral damage, as compared to the sham group, which had normal brain tissue appearance. After 30 min of global cerebral ischemia and then reperfusion, prior studies in line with our findings found that the ischemic group had a strong neutrophil invasion, an expansion of the cytoplasmic space, a reduction in cell density, alterations to the cell structure, and the death of neuronal cells as well as bleeding [69]. In contrast to the sham group, which showed no abnormalities in brain tissue, those who suffered from global cerebral I/R injury showed mild to severe cerebral disability. Verified morphological alterations in brain tissue, such as increased blood vessel congestion, neutrophil infiltration, edema, pyknotic neurons, hemorrhagic areas, and necrotic cells, were observed in rats following 30 min of global cerebral ischemia and reperfusion compared to normal tissue from the sham group. Also, in line with previous studies, dark neuronal degenerations occur when brains are subjected to 30 min of bilateral common carotid artery occlusions [70]. Because telmisartan, ertugliflozin, and omaveloxolone all have neuroprotective effects and may help shield the brain from oxidative damage via their anti-inflammatory and antioxidant properties, this study found that pre-treatment with any of these drugs before ligation of both common carotid arteries decreased the histopathological score caused by CIRI.

The promising neuroprotective effects of telmisartan and ertugliflozin against cerebral ischemia reperfusion injuries and their potent activation of Nrf2/HO-1 molecular signaling pathway encourage us to confirm and validate its therapeutic effectiveness by computational study. Initially molecular docking study was done for telmisartan and ertugliflozin against close regulators of Nrf2 action which are Keap1 within cytoplasm and GSK-3β within nucleus. The molecular docking analysis revealed that ertugliflozin consistently demonstrated stronger binding affinities than telmisartan across both GSK-3β and Keap1 protein targets. For GSK-3β, ertugliflozin exhibited a binding affinity of −8.9163 kcal/mol, significantly more favorable than telmisartan’s − 7.3012 kcal/mol. This stronger binding can be attributed to ertugliflozin’s multiple interactions, including hydrogen bonds with THR B:138, ASP A:260, and ILE B:62, as well as a halogen bond with VAL B:135, at distances ranging from ∼2.7–3.5 Å. In contrast, telmisartan interacted only via a single pi-cation bond with ARG B:141 at ∼2.7 Å. Similarly, with Keap1, ertugliflozin showed a stronger binding affinity (−8.4122 kcal/mol) compared to telmisartan (−7.7412 kcal/mol). Although telmisartan formed a broader network of interactions involving TYR572, ARG415, SER508, ARG483, and PHE478 through hydrogen bonds, salt bridges, and π–π stacking, ertugliflozin maintained comparable interaction types with fewer residues (ARG415, ARG483, TYR572) but at favorable binding distances (∼2.7–3.0 Å). Collectively, these findings suggest that ertugliflozin may have a more stable and thermodynamically favorable interaction with both proteins, making it a potentially more effective inhibitor than telmisartan.

The compounds that were chosen as possible inhibitors of the target based on the results of the docking and post-docking studies were then used to assess the complexes' conformational stability using 100 ns MD simulation [71]. The RMSD ligands are the most stable and fluctuate the least among all complexes. This result suggests that the ligands' mobility inside the protein's active area is minimal. The interactions between the ligands and the protein's active site, on the other hand, persist for 100 ns because it likes to stay in the active site throughout the simulation. Additionally, compared to telmisartan, ertugliflozin has a larger RMSD in both receptor proteins. During runtime, the telmisartan ligand's RMSD with GSK-3β is the most steady and persistent. The RMSD of proteins also stable bound to ligands of four complexes duration the time 100 ns. However, the RMSD of protein Keap1 with both ligands telmisartan and ertugliflozin higher stable as compare to GSK-3β with ligands telmisartan and ertugliflozin. By concluding all the parameters of RMSD and comparing values of both protein and ligand the complex telmisartan-GSK-3β is most stable [39]. The backbone system likewise supports this RMSD value, as shown by the RMSF value. The divergence of the atomic locations from the beginning point is computed using the RMSF. The local changes that occurred to the protein chain's residues and the variations brought on by the ligand's binding were identified using RSMF in order to validate the outcome. The RMSF plot is used to create a plot with peaks. Throughout the simulation, each peak with a high RMSF value denotes a substantial fluctuation value. It is believed that ligand-protein interactions with RMSF values involve fewer stable bonds. The few high peaks may be related to protein’s dynamic domains or they belong to loop regions, which are more flexible than alpha helices or beta sheet. Because of the N-terminal, the RMSF values are greater from beginning to finish [72]. To check for changes in helices or strands, the protein's secondary structural elements (SSE) are monitored during the simulation [73]. Numerous sections of the secondary structure profile show a high proportion of alpha-helices and beta-sheets during the simulation, indicating a well-conserved structural characteristic present throughout the protein. Complexes Ertu-GSK-3β, Ertu-Keap1, Telmi-GSK-3β, and Telmi-Keap1 have total secondary structure elements of 34.84 %, 47.00 %, 35.52 %, and 45.72 %, respectively (Bharatam et al., 2021). All ligands exhibited various interactions during simulation. Multiple contacts were generated at Lys-323, Arg483, Gln-528, Gln104–530, Gln-563, Tyr-5772, and Ser-602 by the complex Ertu-Keap1, whereas interactions were produced at the residues Lys-60, Ile-62, Thr-138, Tyr-140, and Cys-199 by the complex Ertu-GSK-3β. The complex Telmi-Keap1 had Tyr-334, Arg-415, Arg-483, and Tyr-572 residues maintained in line with simulation time, whereas the complex Telmi-GSK-3β created many contacts at Val-61, Ile-62, Arg-141, and Asn-186 across all simulation time. The computational analysis of the potential mechanistic direct interaction of telmisartan and ertugliflozin with tight regulators of the Nrf2 signaling pathway is strengthened by the results of the negative docking score and the stability of complexes over simulation duration of the molecular dynamic study.

## 5 Conclusions

This study demonstrates that omaveloxolone, telmisartan, and ertugliflozin exert significant neuroprotective effects against cerebral ischemia-reperfusion injury in the BCCAO/R rat model. Their actions are primarily mediated through possible activation of the Nrf2/HO-1 pathway and suppression of key negative regulators, including Keap1 and GSK-3β, leading to attenuation of neuroinflammation. Neuroprotective effects obvious in histopathological findings, largely attributable to their anti-inflammatory actions via modulation of NF-kB signaling and affecting levels of inflammatory mediators such as IL-6, TNF-**α**, and MMP-9. These findings highlight the therapeutic potential of Nrf2 modulation as a promising strategy for ischemic stroke management.

## Limitations

This study has certain limitations. While rodent ischemia–reperfusion models provide valuable mechanistic insights, they cannot fully recapitulate the complexity of human stroke pathology, limiting direct clinical translation. The exclusive use of male rats further restricts the generalizability of the findings, as sex-related differences in ischemic outcomes are well documented. Finally, the relatively small sample size (n = 6 per group) may reduce the statistical power of the study.

## Institutional Review Board Statement

The animal study protocol was approved by the Institutional Animal Care and Use Committee (IACUC), University of Kufa (Reg No. 13191 on May 18, 2025.). The authors complied with the ARRIVE 2.0 guidelines

## Funding

The authors declare that no funds, grants, or other support were received during preparation of this manuscript.

## CRediT authorship contribution statement

**Alyassery Yasser J. H.:** Writing – review & editing, Writing – original draft, Methodology, Conceptualization. **Ahsan F. Bairam:** Writing – review & editing, Visualization, Validation, Supervision, Methodology. **Carlos Medina Martin:** Writing – review & editing, Visualization, Validation, Supervision.

## Declaration of Competing Interest

The authors declare that they have no known competing financial interests or personal relationships that could have appeared to influence the work reported in this paper.

## Data Availability

Data will be made available on request.
