# Supplementary material for: Attenuation of cerebral ischemia-reperfusion induced neurotoxicity by telmisartan, ertugliflozin, and omaveloxolone through Nrf2/HO-1 pathway modulation: In vivo and in silico insights
Source: Toxicol Rep. 2025 Nov 19;15:102170. doi: 10.1016/j.toxrep.2025.102170 (PMC12671038; doi:10.1016/j.toxrep.2025.102170)
Supplement: Supplementary file 1 — Supplementary material [file mmc1.docx]

**Nrf2 Immunohistochemistry Optical Images**

*Group 1 (Sham)*


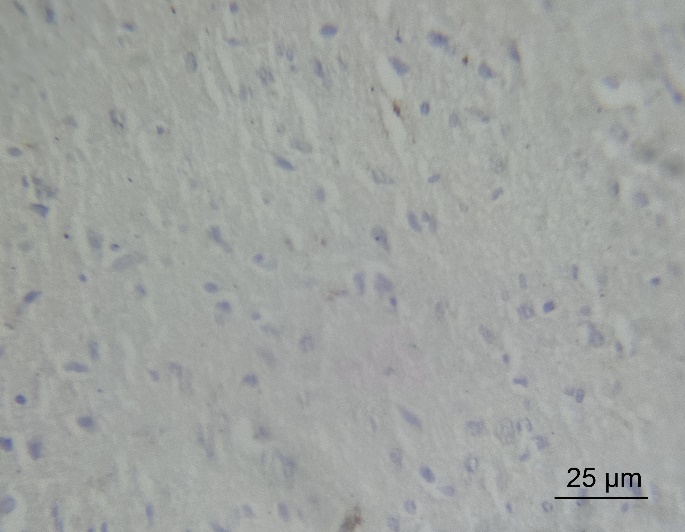

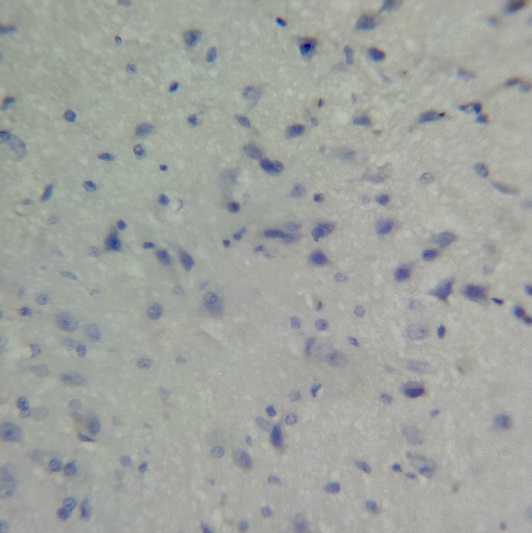


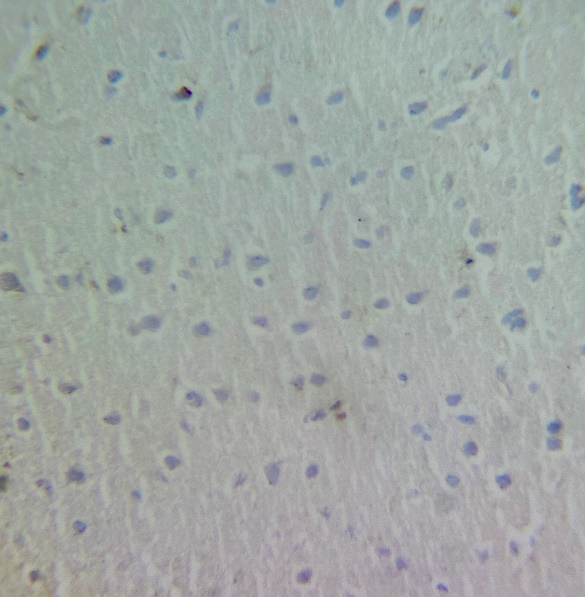

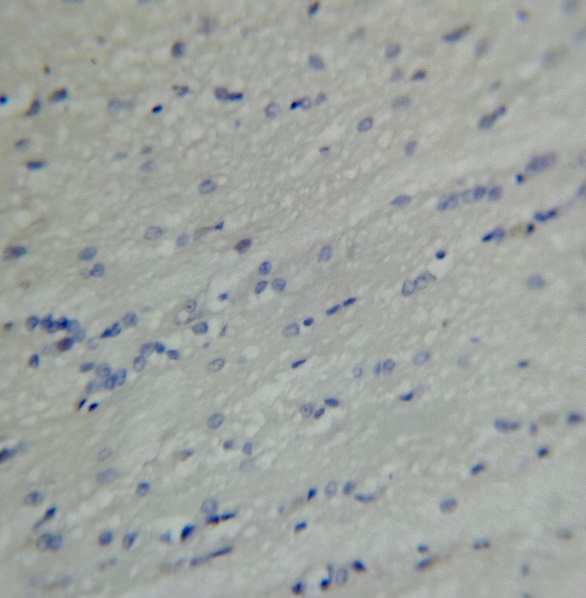


*Group 2 (Control)*


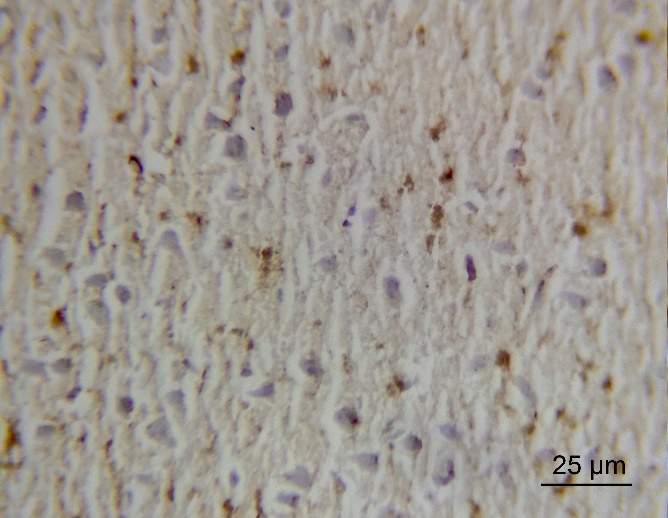

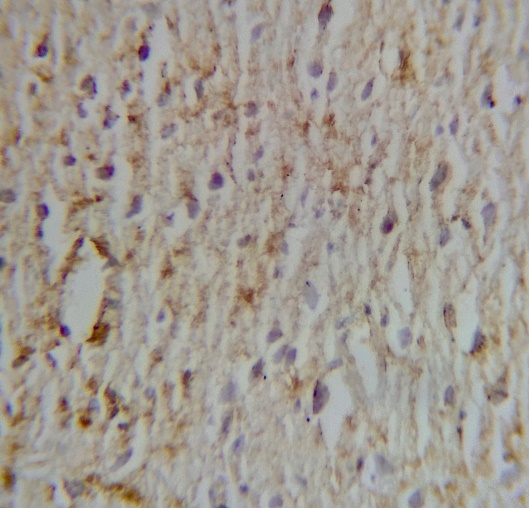

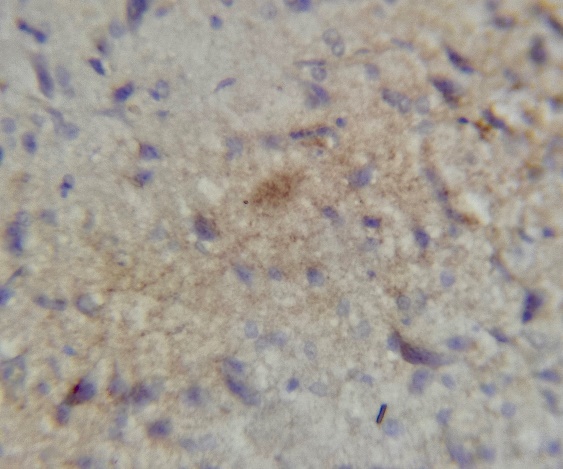

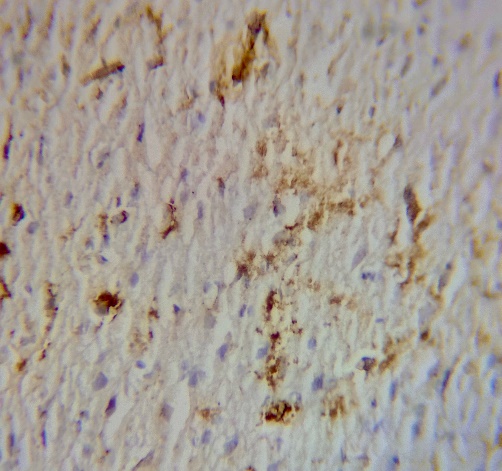


*Group 3 (Vehicle A)*


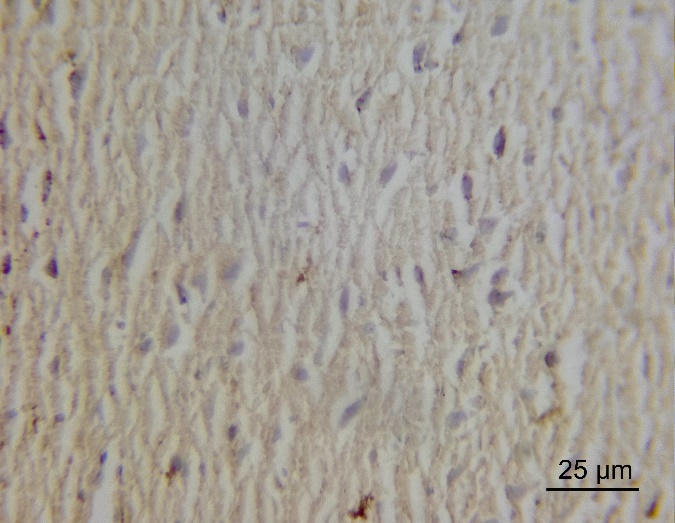

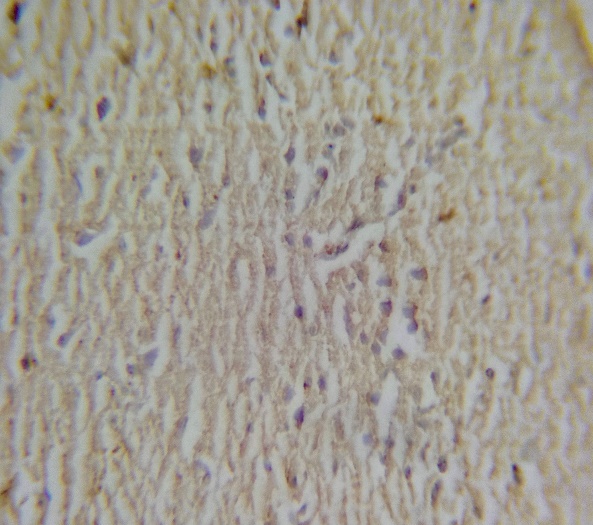


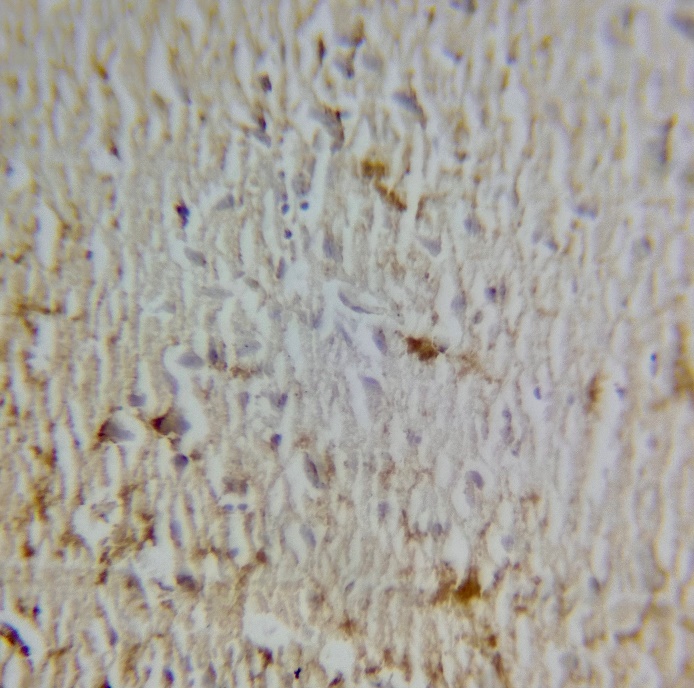

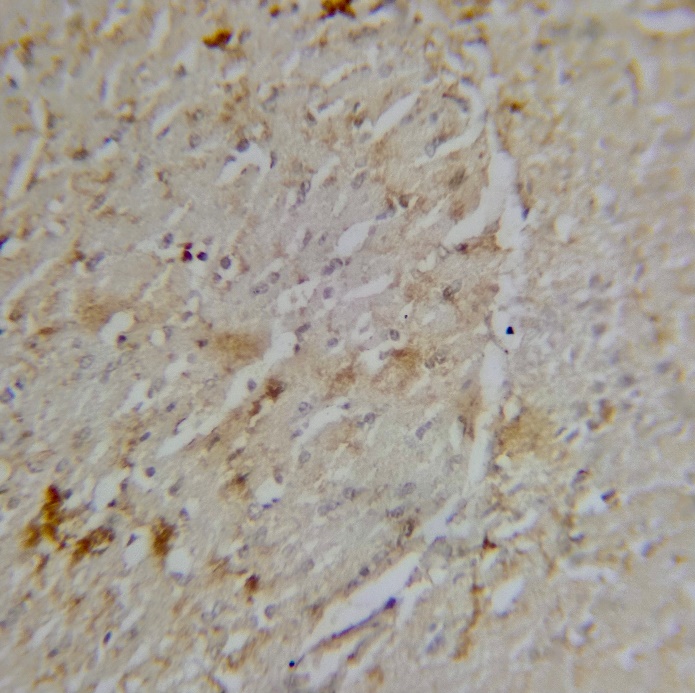


Group 4 (Vehicle B)


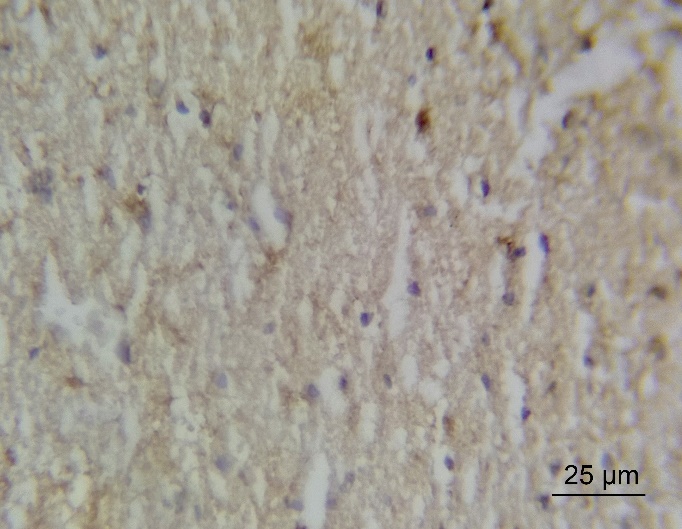

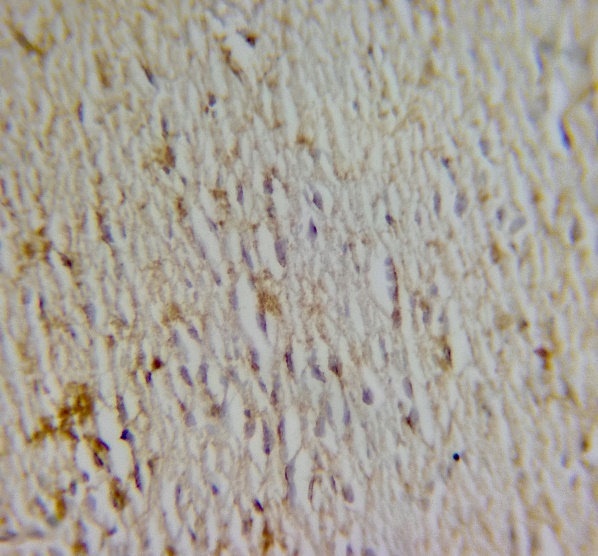


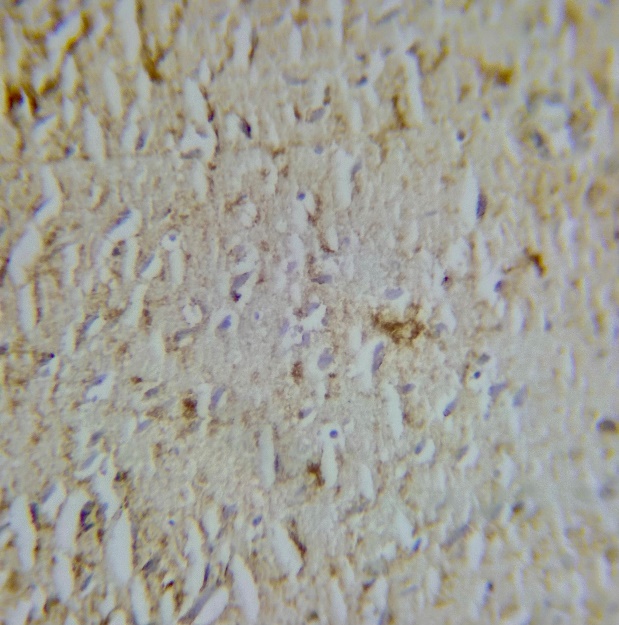


*Group 5 (Telmisartan)*


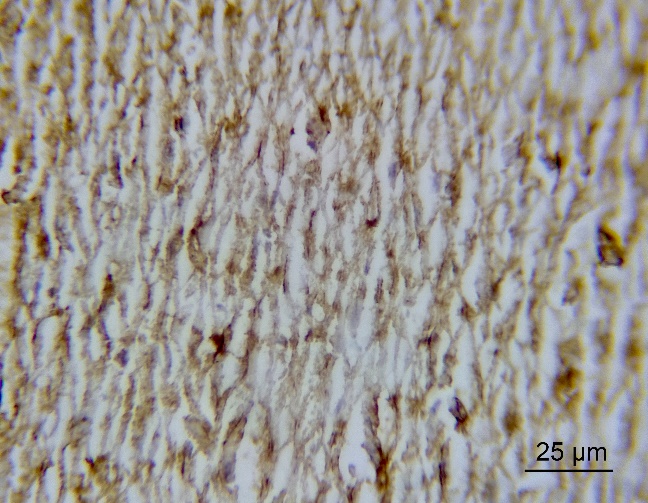

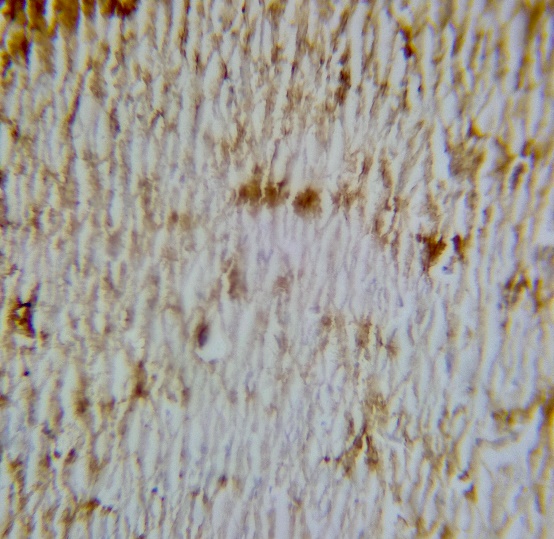


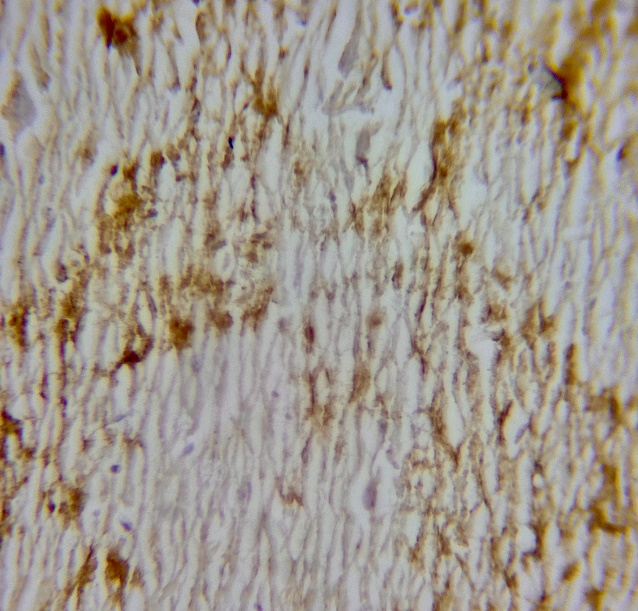

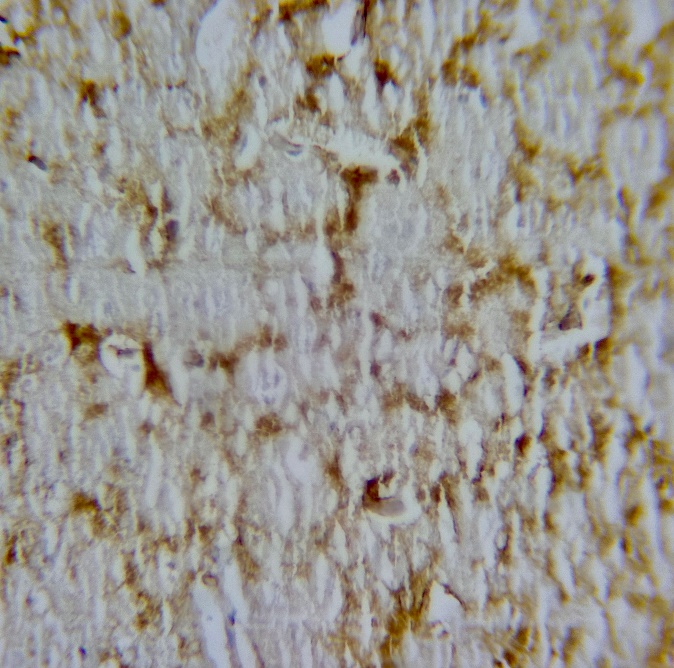


*Group 6 (Ertugliflozin)*


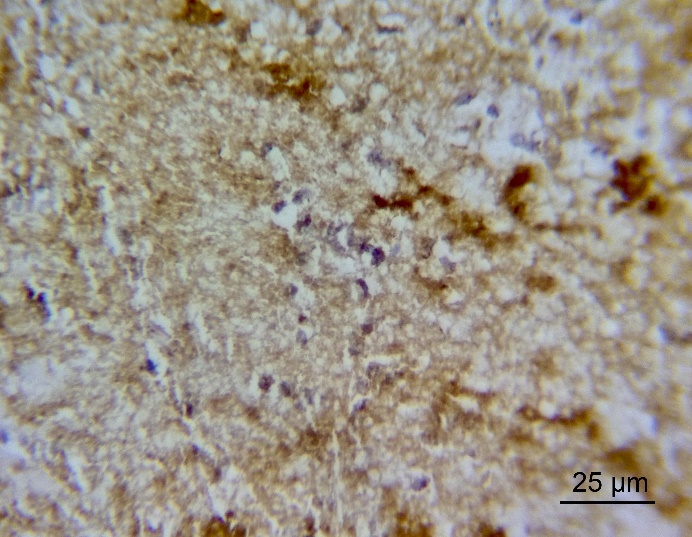

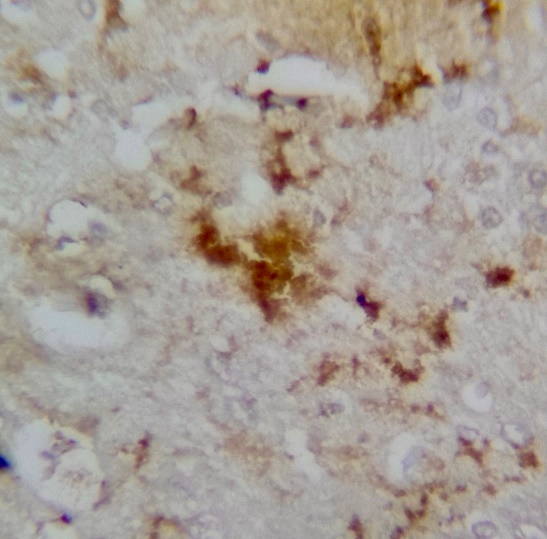


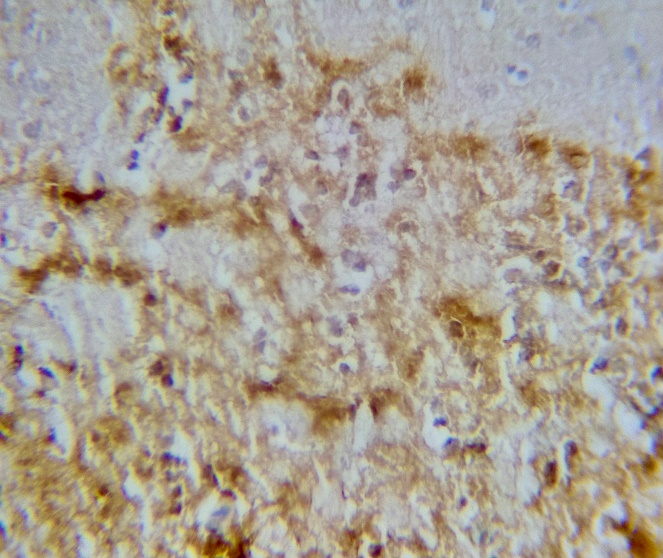

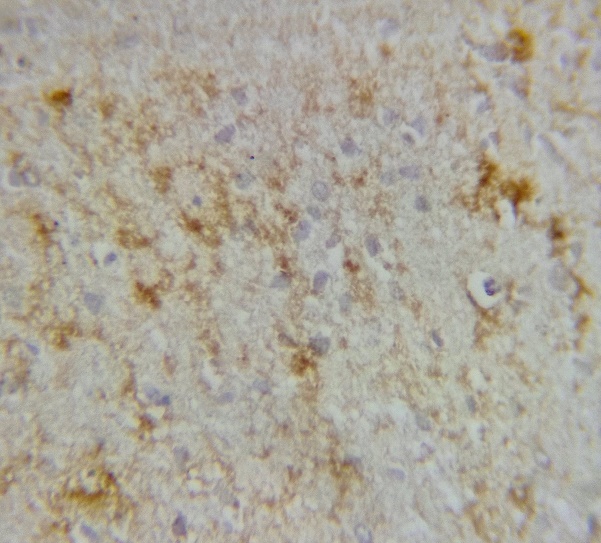


Group 7 (Omaveloxolone)


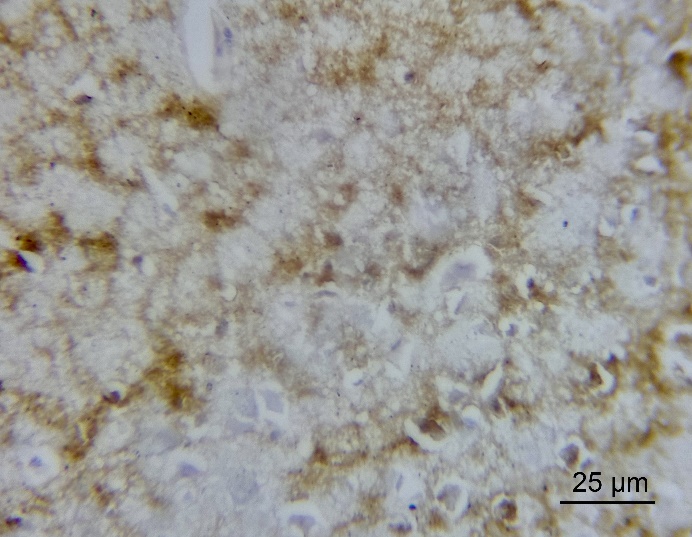

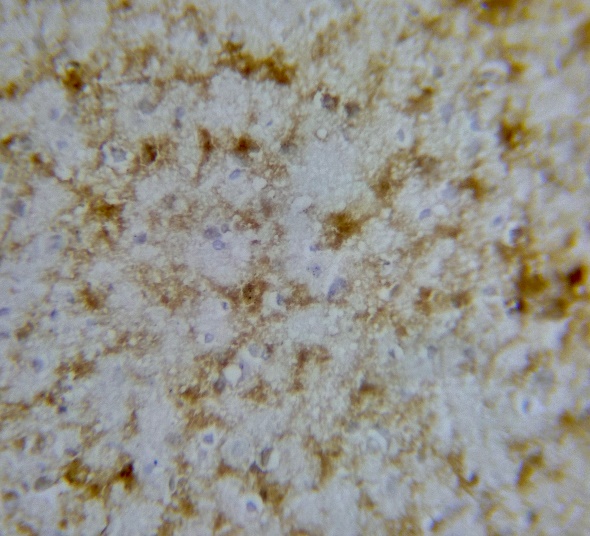


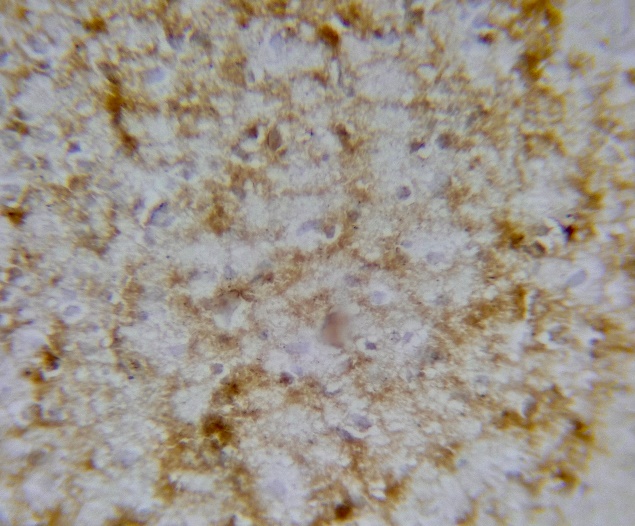

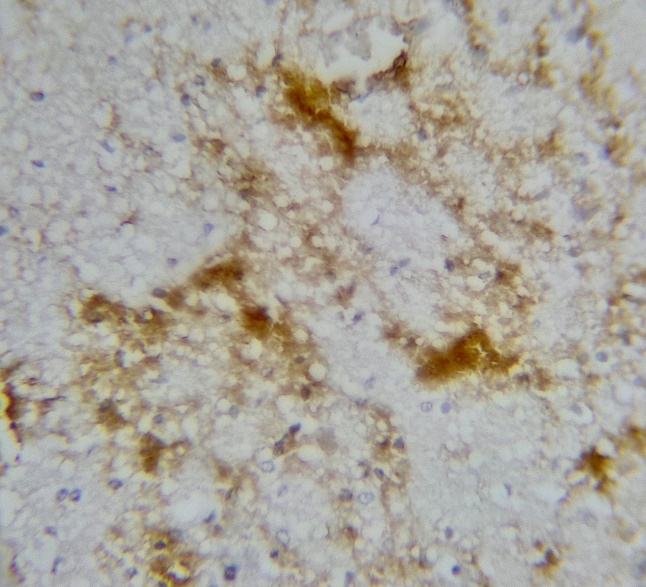


**NF-kB Immunohistochemistry Optical Images**

*Group 1 (Sham)*

*
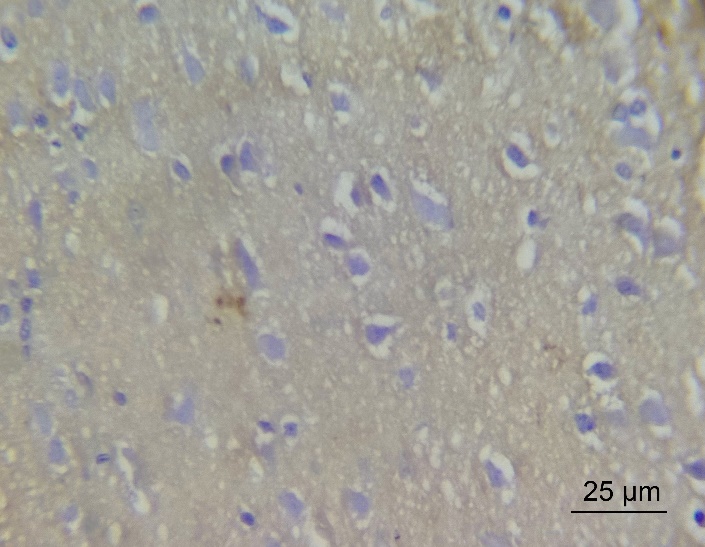

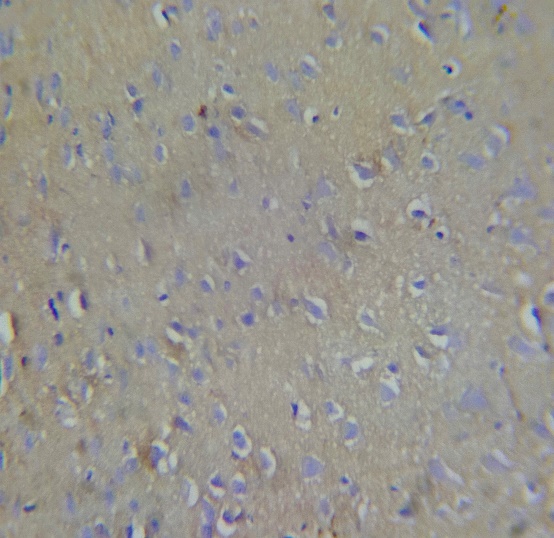
*

*
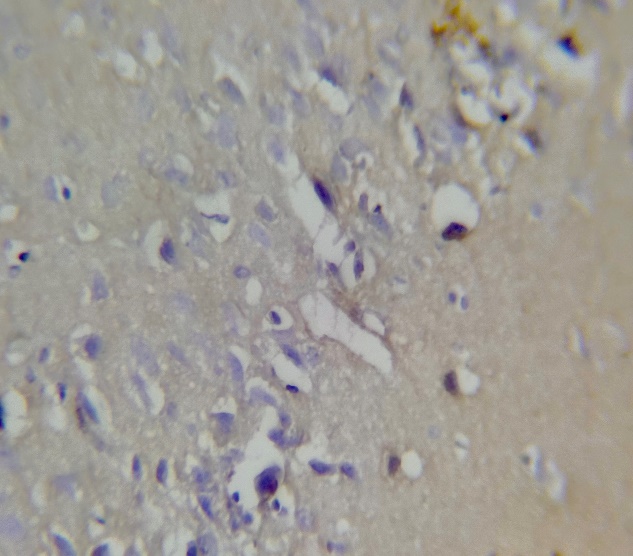

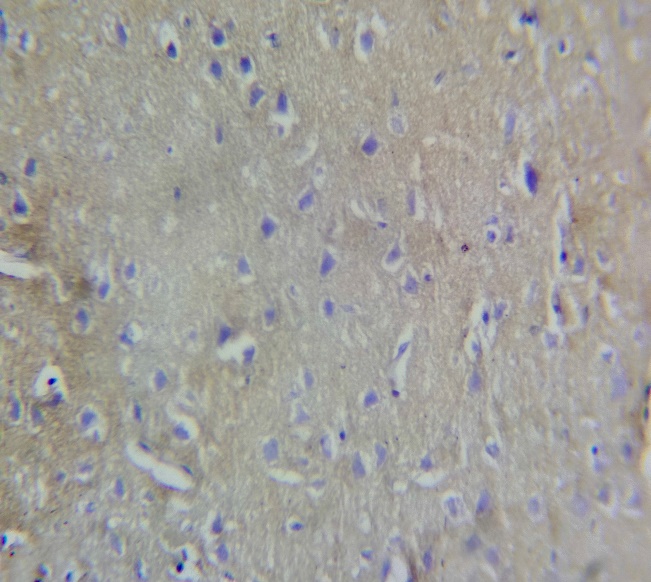
*

*Group 2 (Control)*

*
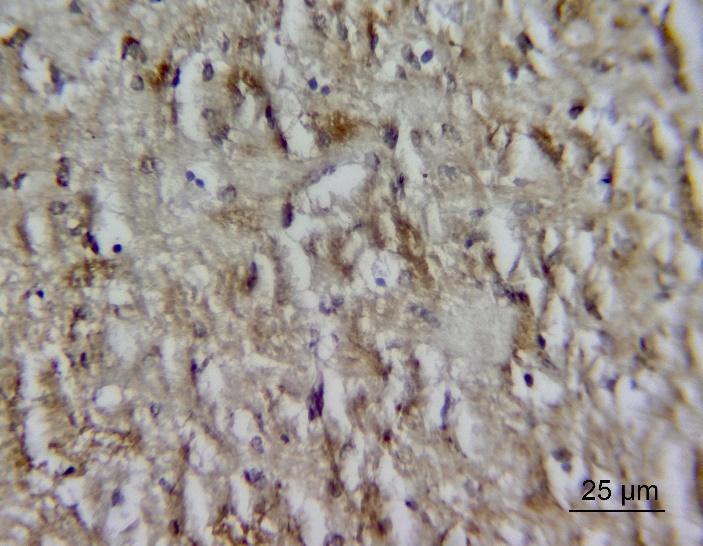

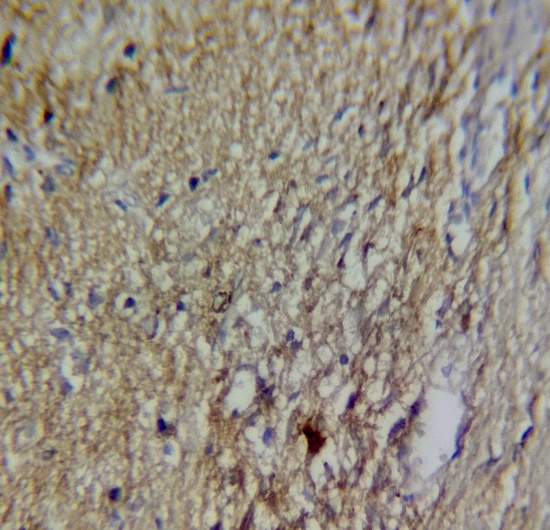
*

*
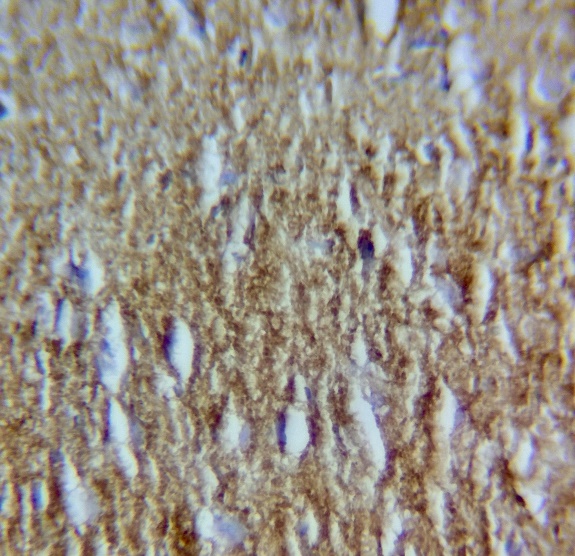

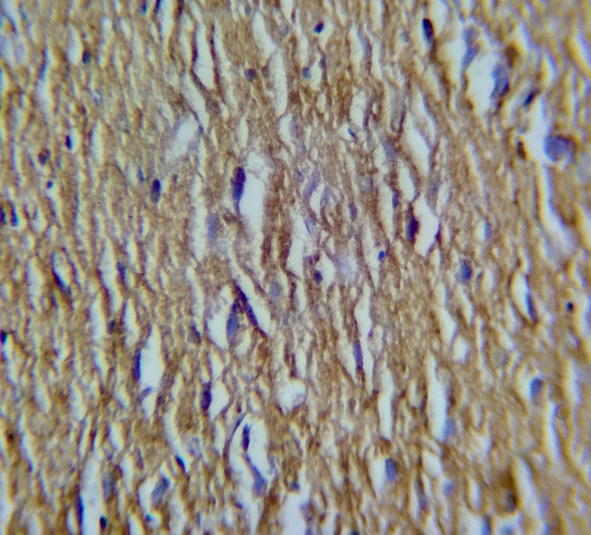
*

*Group 3 (Vehicle A)*

*
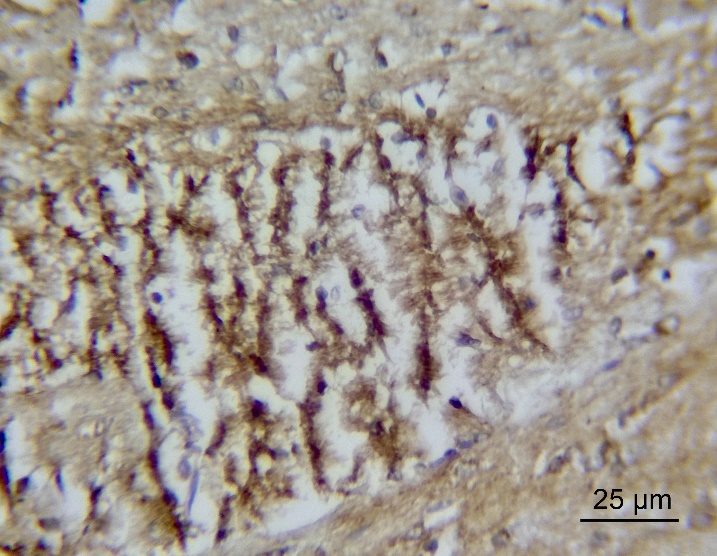

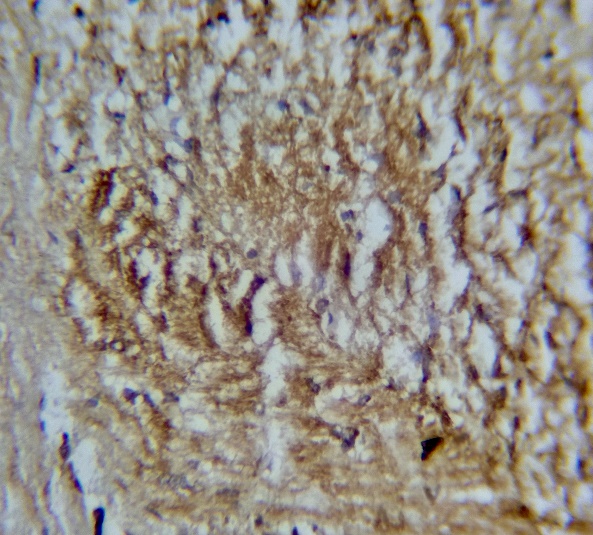
*

*
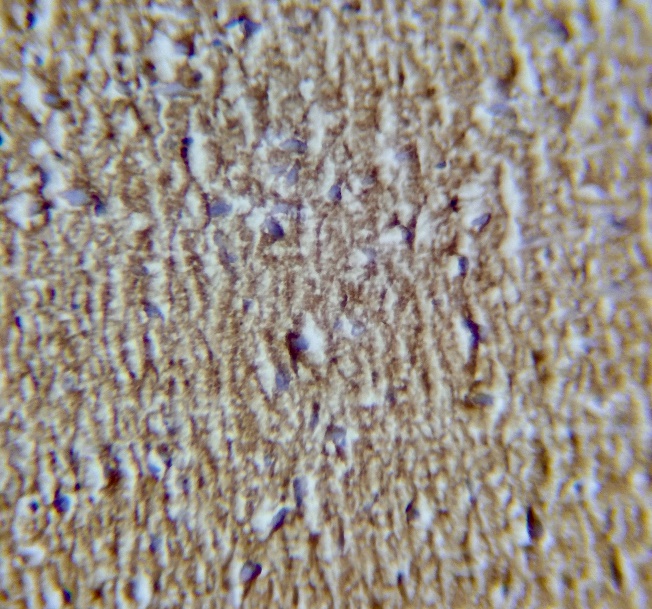

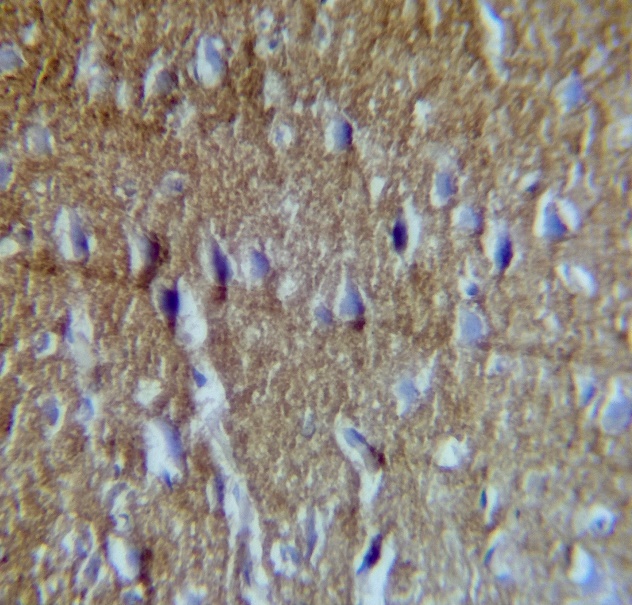
*

*
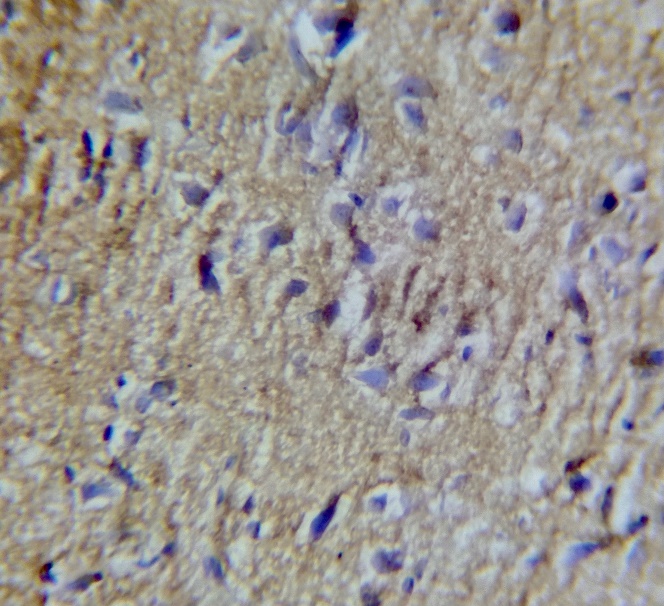
*

*Group 4 (Vehicle B)*

*
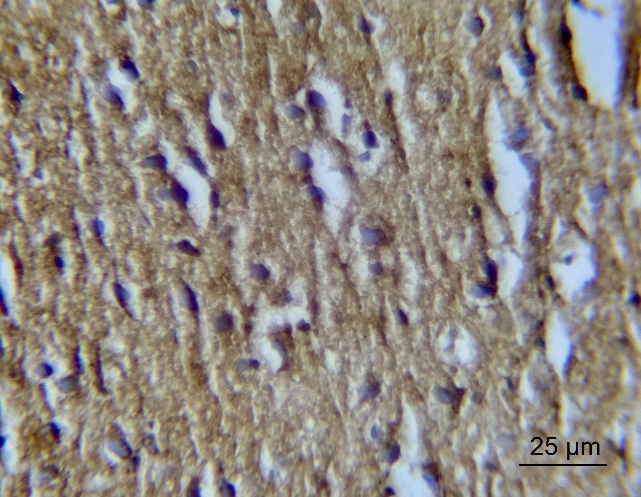

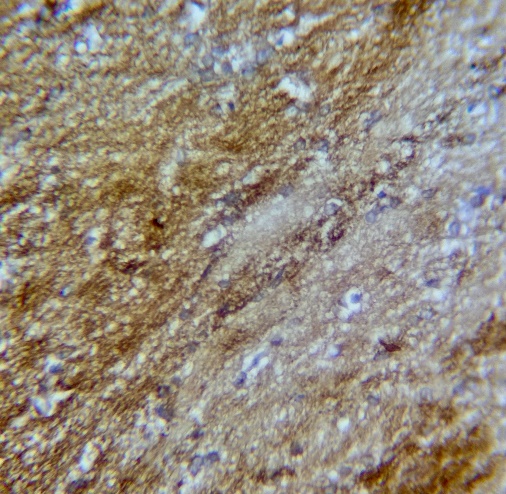
*

*
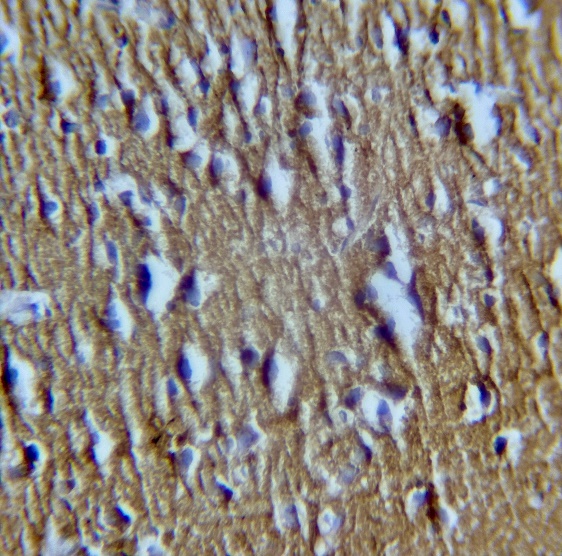

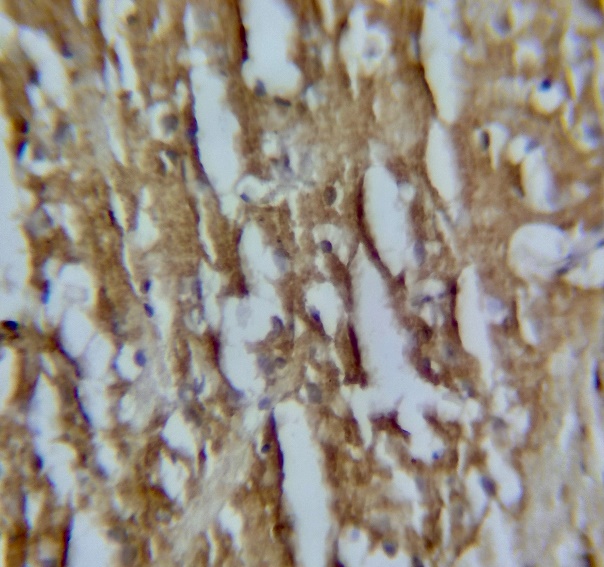
*

*Group 5 (Telmisartan)*

*
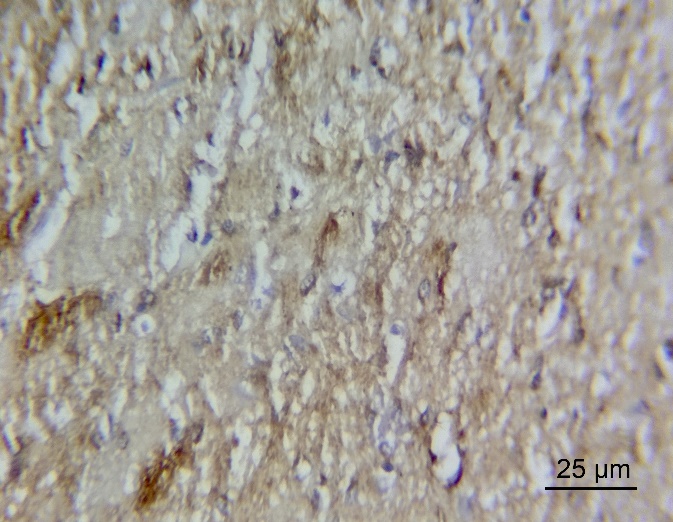

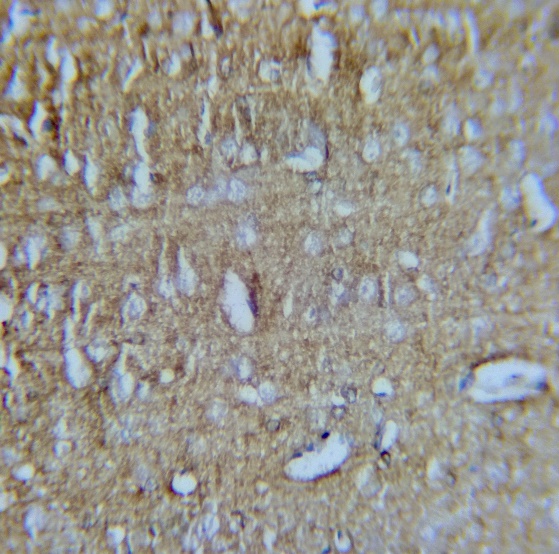
*

*
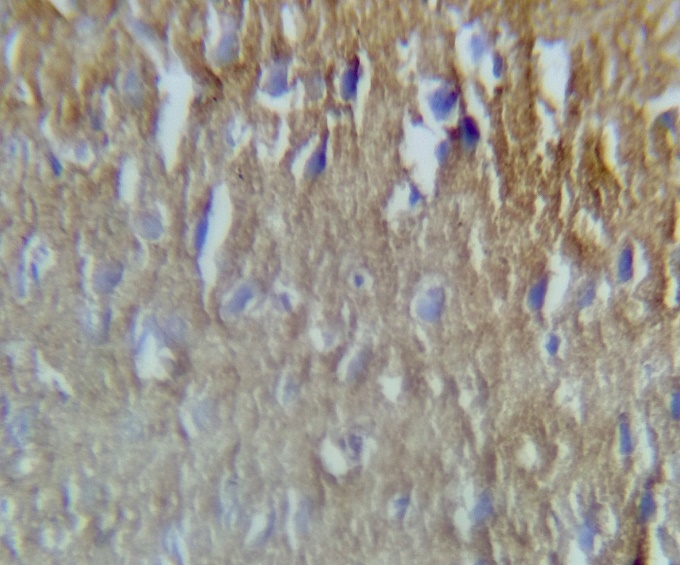

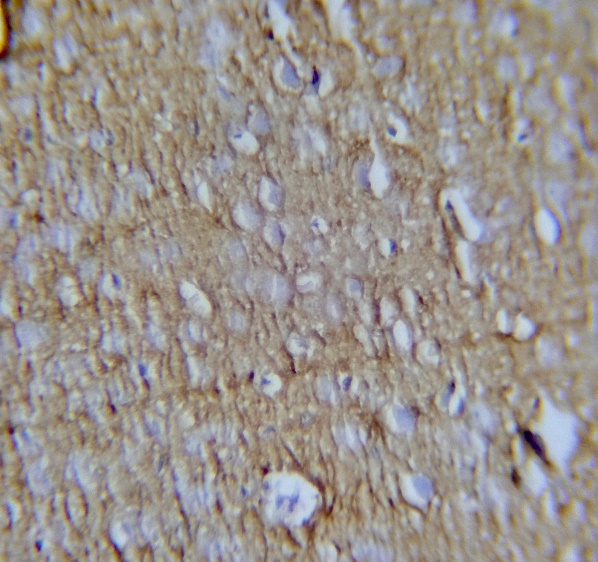
*

*Group 6 (Ertugliflozin)*

*
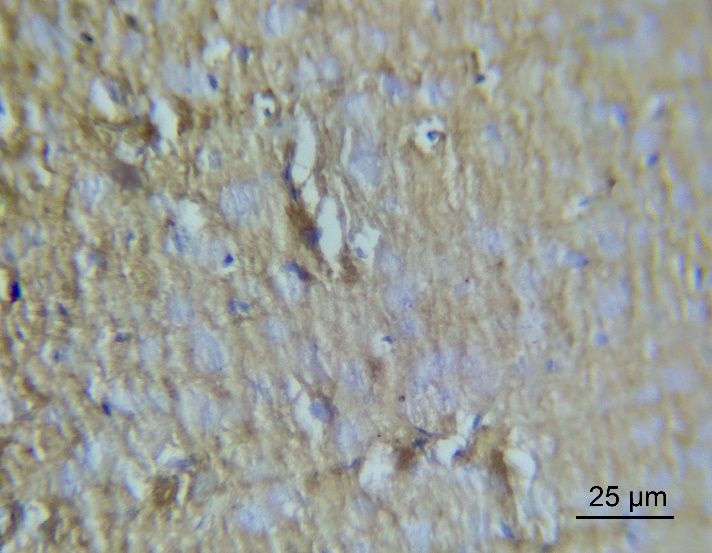

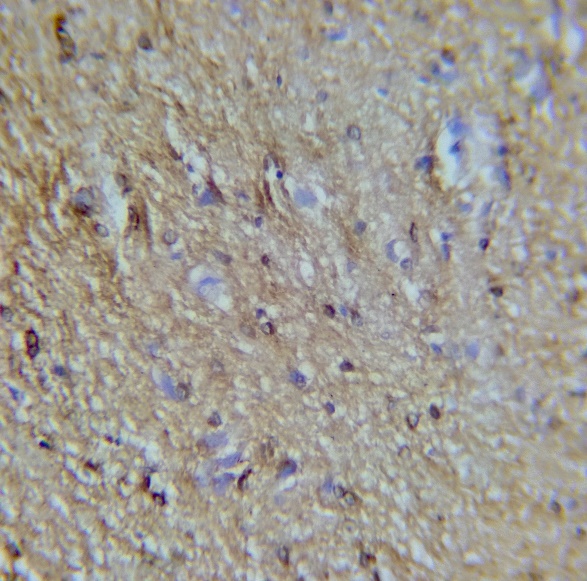
*

*
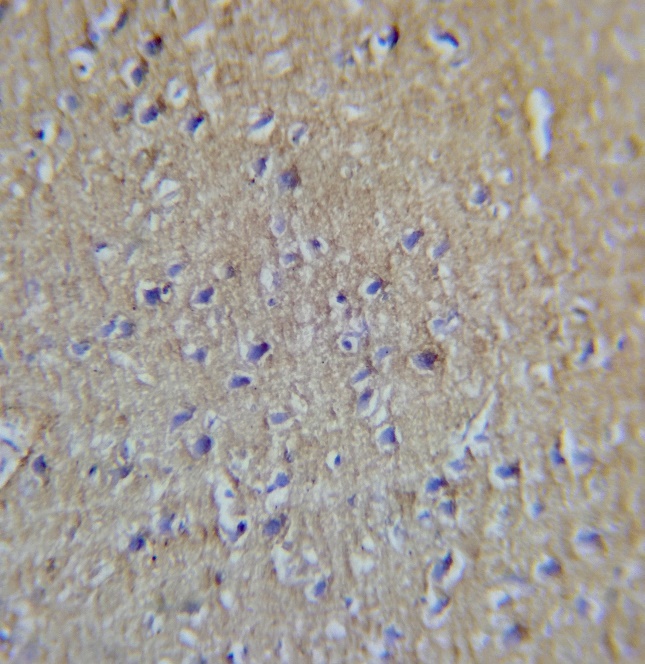

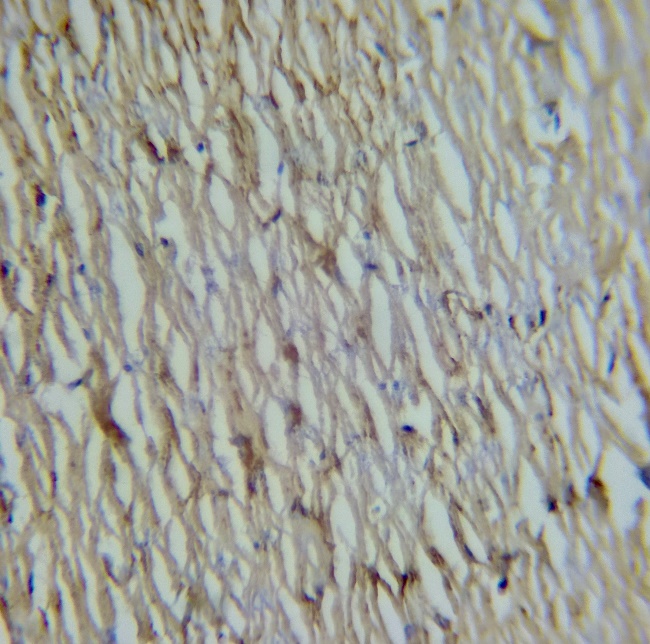
*

*Group 7 (Omaveloxolone)*

*
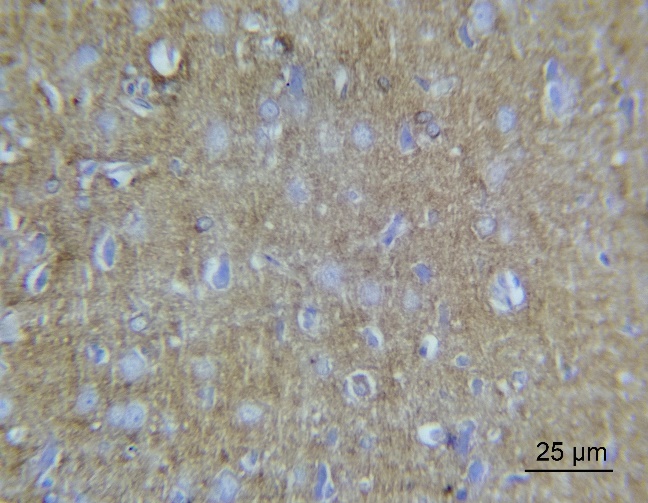

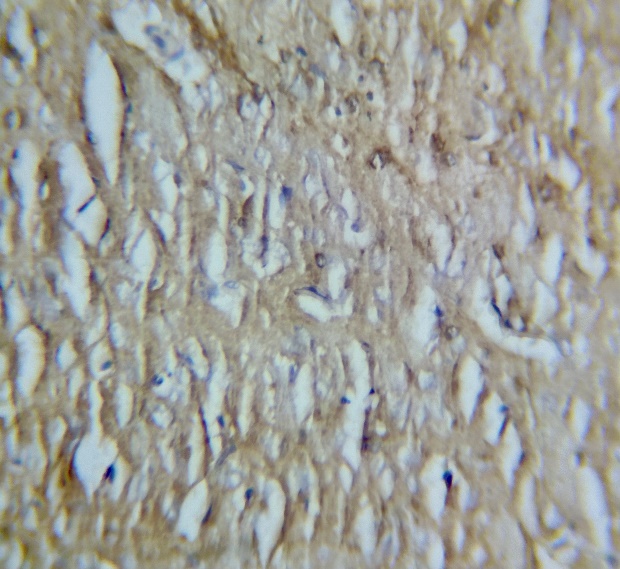
*

*
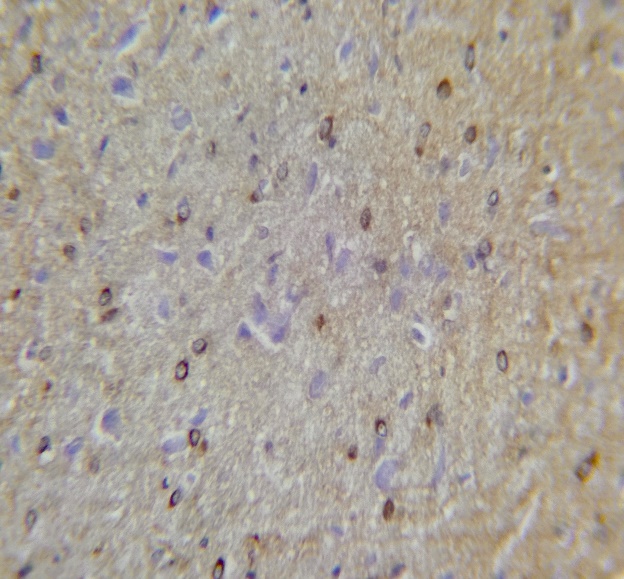

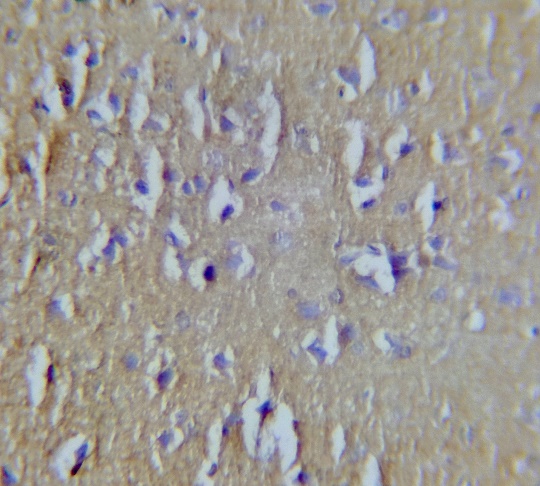
*
